# Supplementary material for: Allosteric regulation and crystallographic fragment screening of SARS-CoV-2 NSP15 endoribonuclease
Source: Nucleic Acids Res. 2023 Apr 28;51(10):5255–70. doi: 10.1093/nar/gkad314 (PMC10250223; doi:10.1093/nar/gkad314)
Supplement: gkad314_Supplemental_Files [file gkad314_supplemental_files.zip › Supplementary datasets.zip!/224/Refine_9/01-BUSTER/Cycle-5/LIST.html]

Refining

**BUSTER 2.X**: A suite of programs for macromolecular structure refinement by maximum-likelihood and Bayesian methods  
Copyright © 1992-2020 Global Phasing Ltd.  
All rights reserved.  
Authors: G. Bricogne, E. Blanc, M. Brandl, C. Flensburg, P. Keller, W. Paciorek, P. Roversi, O. Smart, C. Vonrhein, T. Womack  

Please reference use of the software as:

- BUSTER-TNT 2.X, Global Phasing Ltd, Sheraton House, Cambridge CB3 0AX, UK

Version: 2.10.3 for linux64-ifort made on May 20 2020 at 01:14:34  
Licenced to: Diamond Light Source, Scientific Software  

```
---


### Parsing of the BUSTER cardfile

TITLE : autoBUSTER refinement

   Cell information

   Symmetry information

 Using CCP4 X-ray form factors.

   Missing structure chemical composition

 Using ATOM block for chemical composition.


   Shell information

### Parsing of the TNT cardfile


---


### Reading observations file

 Number of reflexions in input:  58343

      Number of accepted reflexions:  52749

      Number of rejected reflexions:      0

   Preparing complete.mtz containing:      58207 reflexions


   Reflexions in test set:            2702

   Reflexions in working set:        50047


---


### 1. Generating initial model

 2.10 * Shannon sampling rate. NX NY NZ:   288  288  240


  Effective Shannon rates are:    2.29    2.29    2.55


   Allocated BUFFER of size:     76.465 Mb.

   Initial Geometry Information


 No missing atoms declared


   Summary of model volumes

 No missing atoms declared: no prior computed


 Computing bulk solvent model scattering


 NCSMASK PDB radius for solvent mask:     2.15 Angstrom

 running PDBSET (setting cell and spacegroup P1) ... done
 running NCSMASK [1] (creating mask, SMOOTH 1.04842) ... done
 running MAPDUMP (to check if reduction to unit cell is needed) ... done
 running MAPMASK (converting mask to map) ... done

   Generation of babslv by theta-filter

   Electron density in frg mask:  0.420 Å-1

 FOBS  put on rough absolute scale by Wilson plot :
           K        =  0.66646E+02
           B        =     -5.851

 

### Cycle 0001: MaxLik scaling and refinement


   MaxLik Scaling Cycle 0001

   Wilson plot

   Statistics in resolution bins just after scaling
```

|  |  |
| --- | --- |
|  | - Rxpct factors plot- Rxpct factors table- <Log-Lik Gain>- Ln Sigma A- Correlation Coefficients |

```
   <Log-Likelihood> of null-hypothesis

Structure Factor Amplitudes


   Average Figure of Merit

 Switching off refinement of overall temperature factor


 Added B =    -0.2211 Å2 to all atoms in fragment


 Added B =    -0.2211 Å2 to B_SOLV

```
gelly refinement module
 Authors (2004-2009) O. S. Smart, C. Flensburg, W. Paciorek, C. Vonrhein, 
                     M. Brandl, T. O. Womack and G. Bricogne
         (2010-2011) O. S. Smart, C. Flensburg, W. Paciorek, C. Vonrhein, 
                     T. O. Womack and G. Bricogne
         (2012-2014) O. S. Smart, C. Flensburg, W. Paciorek, C. Vonrhein, 
                     and G. Bricogne
         (2015-2020) G. Bricogne, C. Flensburg, W. Paciorek, C. Vonrhein
 Copyright © 2004-2020 Global Phasing Ltd. 
 All rights reserved.
 gelly module compilation date May 20 2020 01:11:20
 
 gelly uses routines from TNT geometry. References: 
     Tronrud, D. E., Ten Eyck, L. F., Matthews, B. W. (1987)
     "An Efficient General-Purpose Least-Squares Refinement Program 
     for Macromolecular Structures". Acta Crystallogr A, 43:489-501.
     Tronrud, D. E. (1997). "The TNT Refinement Package", 
     in Macromolecular Crystallography, Part B, Eds Charlie Carter 
     and Robert Sweet, Methods in Enzymology, 277:306-319.
 wildcard matching function by Julian Robichaux http://www.nsftools.com

 No -random_seed flag found. Random number stream initialised using:
2147483562
2147483398

 Control flags set to:  -autoncs -hydrogens_input_occ2zero \
                     -hydrogens_output_occ2heavy -screen \
                     100 -screen_sigma \
                     3.0 -keeppdb \
                     1000000 -taomonitor_verbose \
                     2 
 

 ############### Starting setup via TNT routines

 Setting up tnt routines including license check:

                   TNT Stereochemistry Program
 Version: 5.22.3.0
 Licence for Diamond Light Source, Scientific Software is valid
 
 Using TNT geometry routines to load the tnt control file: gelly.tnt
 Using TNT routines to load initial coordinates from file fragment.cor
 Expecting 6506 atoms

 Found     2197 "NOTE BUSTER_RESIDUE_ATOM_DATA" cards
 Have copied information for    6506 atoms from TNT to gelly's store 
 
 The TNT WEIGHT cards used by this run:
 -----WEIGHT       BOND     2.0
 -----WEIGHT       ANGLE    2.0
 -----WEIGHT       IMPROPER 0.0
 -----WEIGHT       TORSION  2.0
 -----WEIGHT       PSEUDO   0.0
 -----WEIGHT       TRIGONAL 2.0
 -----WEIGHT       PLANE    5.0
 -----WEIGHT       CONTACT  5.0
 -----WEIGHT       BCORREL  10.0
 -----WEIGHT       CHIRAL   5.0
 -----WEIGHT       NCS      50.0
 -----WEIGHT       IDEAL    4.0
 -----WEIGHT       RFACTOR  7.45
 
 Dictionary versions picked up from NOTE BUSTER_RESTRAINT_DICT cards as: 
 ------ protgeo_eh99.dat (V1.6.4.17) 20191001  STANDARD AMINO ACID DICTIONARY. BONDS AND ANGLES FROM ENGH AND HUBER EH99. OTHER VALUES BASED ON PREVIOUS TNT OR TAKEN FROM CCP4. INCLUDES HYDROGEN ATOMS.
 ------ exoticaa.dat (V1.3.4.15) 20200327  COLLECTION OF NON-STANDARD AMINO ACIDS, MAINLY EH91 WITHOUT IDEAL DISTANCE INFO
 ------ nuclgeo.dat (V1.13.4.8) 20190909
 ------ bcorrel.dat (V1.15.10.2) 20190616
 ------ contact.dat (V1.15.12.10) 20191121
 ------ idealdist_contact.dat (V1.3.4.12) 20191001  IDEAL-DISTANCE CONTACT TERM DATA AS USED IN PROLSQ. VALUES USED HERE ARE BASED ON DATA HARVESTING OF PDB AS OF 20190109.
 ------ restraints for LIG (Unknown) from cif dictionary Z927400026.cif
 ------ restraints for CIT (CITRIC ACID) from cif dictionary CIT.cif; generated by GRADE_PDB_LIGAND 1.2.12 (October 4 2016) using MOGUL 1.7.2(RC1), CSD as538be, with quantum mechanics RM1 ((DatabaseID PDB-CIT))
 ------ assume.dat (V1.9.4.2) 20180705
 
 Have picked up spacegroup name for pdb output as 'P 63      '
 Setting up Chain/Residue arrays
 
 Have command line specification -autoncs*
     Have found       1 NCS relations
     Pairwise matching residue number and type between chains: 
                    A        B        S        W    
          A         .       99%       -        -   
          B                  .        -        -   
          S                           .        -   
          W                                    .   
       Table key--                                                       
          all   : two chains have 100% match and same number of residues 
         multi  : ditto and they contribute to a multi chain ncs cluster 
          ***%  : two chains have >80% matching residue number/type      
           -    : chains appear to be unrelated                          
      chain A     have identified ncs to     1 other chains. So will weight each autoncs by (       6.666/#)=      6.6660
      chain B     have identified ncs to     1 other chains. So will weight each autoncs by (       6.666/#)=      6.6660
      chain S     have identified ncs to     0 other chains. 
      chain W     have identified ncs to     0 other chains. 
     For .Gelly cards produced see below - after pruning
 
 Setting up BUSTER_SET groups
 Have found    89 BUSTER_SET cards
 Now silently applying automatic/dictionary set definitions. For a verbose listing use '-verbose_set' flag.
 Applying user defined set definitions:
 =====NOTE BUSTER_SET ncsautoXcld = Water --> set 'ncsautoXcld' now has   966 atoms 
 =====NOTE BUSTER_SET ncsautoABset = Chain_A \ ncsautoXcld --> set 'ncsautoABset' now has  2770 atoms 
 Have defined    49 sets: 
      set:                 'All' has   6506 atoms in   1666 residues - automatically defined
      set:               'Empty' has      0 atoms in      0 residues - automatically defined
      set:            'ExoticAA' has      0 atoms in      0 residues - defined by dictionary file
      set:           'ExoticNUC' has      0 atoms in      0 residues - defined by dictionary file
      set:              'NotHET' has   5514 atoms in    698 residues - defined by dictionary file
      set:             'Chain_A' has   2770 atoms in    350 residues - automatically defined
      set:             'Chain_B' has   2770 atoms in    350 residues - automatically defined
      set:             'Chain_S' has    567 atoms in    567 residues - automatically defined
      set:             'Chain_W' has    399 atoms in    399 residues - automatically defined
      set:               'Water' has    966 atoms in    966 residues - defined by dictionary file
      set:          'StdProtein' has   5514 atoms in    698 residues - defined by dictionary file
      set:             'Protein' has   5514 atoms in    698 residues - defined by dictionary file
      set:                'Back' has   2794 atoms in    698 residues - defined by dictionary file
      set:                'Side' has   2720 atoms in    650 residues - defined by dictionary file
      set:        'RnaCanonical' has      0 atoms in      0 residues - defined by dictionary file
      set:        'DnaCanonical' has      0 atoms in      0 residues - defined by dictionary file
      set:     'RnaDnaCanonical' has      0 atoms in      0 residues - defined by dictionary file
      set:     'DnaRnaCanonical' has      0 atoms in      0 residues - defined by dictionary file
      set:         'RnaExtended' has      0 atoms in      0 residues - defined by dictionary file
      set:         'DnaExtended' has      0 atoms in      0 residues - defined by dictionary file
      set:      'RnaDnaExtended' has      0 atoms in      0 residues - defined by dictionary file
      set:      'DnaRnaExtended' has      0 atoms in      0 residues - defined by dictionary file
      set:                 'Dna' has      0 atoms in      0 residues - defined by dictionary file
      set:                 'Rna' has      0 atoms in      0 residues - defined by dictionary file
      set:              'RnaDna' has      0 atoms in      0 residues - defined by dictionary file
      set:              'DnaRna' has      0 atoms in      0 residues - defined by dictionary file
      set:             'Nucleic' has      0 atoms in      0 residues - defined by dictionary file
      set:             'Polymer' has   5514 atoms in    698 residues - defined by dictionary file
      set: 'BufferWeichenberger' has     13 atoms in      1 residues - defined by dictionary file
      set:    'BufferAdditional' has      0 atoms in      0 residues - defined by dictionary file
      set:              'Buffer' has     13 atoms in      1 residues - defined by dictionary file
      set:         'MetalAlkali' has      0 atoms in      0 residues - defined by dictionary file
      set:  'MetalAlkalineEarth' has      0 atoms in      0 residues - defined by dictionary file
      set:     'MetalTransition' has      0 atoms in      0 residues - defined by dictionary file
      set: 'MetalPostTransition' has      0 atoms in      0 residues - defined by dictionary file
      set:    'MetalLanthanides' has      0 atoms in      0 residues - defined by dictionary file
      set:               'Metal' has      0 atoms in      0 residues - defined by dictionary file
      set:         'SugarAlphaD' has      0 atoms in      0 residues - defined by dictionary file
      set:         'SugarAlphaL' has      0 atoms in      0 residues - defined by dictionary file
      set:               'Sugar' has      0 atoms in      0 residues - defined by dictionary file
      set:               'Other' has     26 atoms in      2 residues - defined by dictionary file
      set:            'Hydrogen' has      0 atoms in      0 residues - defined by dictionary file
      set:         'nonHydrogen' has   6506 atoms in   1666 residues - defined by dictionary file
      set:             'OccZero' has      0 atoms in      0 residues - defined by dictionary file
      set:          'OccNonZero' has   6506 atoms in   1666 residues - defined by dictionary file
      set:             'OccFull' has   6461 atoms in   1663 residues - defined by dictionary file
      set:          'OccNonFull' has     45 atoms in      3 residues - defined by dictionary file
      set:         'ncsautoXcld' has    966 atoms in    966 residues - user specified
      set:        'ncsautoABset' has   2770 atoms in    350 residues - user specified
 No command line arguments '-occ_allhet' or '-occ_restyp' so will not setup automated occcupancy refinement
 
 Interpreting CONSTANT and FREE cards: 
 =====CONSTANT     OCC
 Have found and interpreted      1 CONSTANT cards in the TNT input. 
 Before any COMBINE - Number of atoms with fixed XYZ =     0
 Before any COMBINE - Number of atoms with fixed B   =     0
 Before any COMBINE - Number of atoms with fixed OCC =  6506
 Before any COMBINE - Number of atoms with fixed BIJ =   966
                            cf total number of atoms =  6506
 
 There are no COMBINE cards to be found in the TNT card input 
 
 There are no NOTE BUSTER_COMBINE_RES cards to be found in the TNT card input 

 ############### Working out full connectivity list


 ############### Working out equivalent atoms


 Residue with most atoms given below:
        A|58:N(TRP)         
        A|58:CA(TRP)        
        A|58:CB(TRP)        
        A|58:CG(TRP)        
        A|58:CD1(TRP)       
        A|58:NE1(TRP)       
        A|58:CE2(TRP)       
        A|58:CD2(TRP)       
        A|58:CE3(TRP)       
        A|58:CZ3(TRP)       
        A|58:CH2(TRP)       
        A|58:CZ2(TRP)       
        A|58:C(TRP)         
        A|58:O(TRP)         


 Have no '-equiv_check_plus' argument - so will take atom type into
 account when checking for equivalence of otherwise identical atoms.
 

 ############### Looking at dihedrals


 ############### Looking at chirals

 NOTE BUSTER_CHIRAL_ANGLECUT card found - read value=    -31.00 degs from it
 NOTE BUSTER_CHIRAL_SIGMA card found - read value=      1.70 degs from it
 RANGE allowed for B variables in refinement:   from      3.00000 to    300.00000

 ############### Preparing for PDB output


 ############### Processing extra restraints explicitly defined

 No 'NOTE BUSTER_TARGET' cards or -target?? args found (used to read coordinates files for soft ncs to external)
 No 'NOTE BUSTER_OCCSUM'     cards found - so not using sum of occupancy restraints
 No 'NOTE BUSTER_DISTANCE'   cards found - so not setting up any additional atom-atom distance restraints
 No 'NOTE BUSTER_UTILANGLE'  cards found - so not setting up any additional restraints on any angle
 No 'NOTE BUSTER_UTILTOR'    cards found - so not setting up any additional sinusoidal restraints on torsions
 
 --- Have some similarity definitions 
     Similarity restraints on RMSD use superposition routines from: 
     Coutsias, E.A., Seok, C., Dill, K.A.(2004)
     "Using quaternions to calculate RMSD", J. Comput. Chem., 25:1849-1857 

 Have found    1 'NOTE BUSTER_SIM_DEFINE' cards 
     Maximum number of chains involved in any SIM group (excluding the template)=     1


 ########## Interpreting card: NOTE BUSTER_SIM_DEFINE ncsautoAB ncsautoABset B
     Number of atoms in template set=  2770
     Number of related chains (other than template) in SIM set=    1
     List of chains to be restrained (excluding template)=   B   
     Pairwise RMSD, XYZ in Angstroms, for SIM group: ncsautoAB
                 A         B   
     A         -----     ----- 
     B         0.563     ----- 
     Overall RMSD,XYZ for the SIM group is     0.563 Angstroms
     Pairwise rms deviation of [individual B's minus their set/chain average] in Angs**2
                 A         B         Bavg
     A         -----     -----     79.03
     B         18.85     -----     63.16
     Overall RMSD,B   for the SIM group is    18.849 Angs**2
     The number of common atoms for the above fits:
                 A         B   
     A         -----     ----- 
     B          2757     ----- 
     (compared to the template set that has    2770 atoms in it)
     List of atoms that are absent in one or more chains for this group: 
                                    A    B   
                    C1      401     +    -
                    O1      401     +    -
                    O2      401     +    -
                    C2      401     +    -
                    C3      401     +    -
                    O7      401     +    -
                    C4      401     +    -
                    C5      401     +    -
                    O3      401     +    -
                    O4      401     +    -
                    C6      401     +    -
                    O5      401     +    -
                    O6      401     +    -
     (key  +  atom present for this chain 
           -  atom absent  for this chain)
 transforms for each of the superpositions orthogonal matrix r11 r12 r13 r21 r22 r23 r31 r32 r33 tx ty tz (PDBSET syntax): 
TRANS_AB -0.877149 -0.480050 -0.012721 -0.480076  0.877226 -0.001125  0.011699  0.005120 -0.999918    33.917    32.989    15.326
 reverse transforms 
TRANS_BA -0.877149 -0.480076  0.011699 -0.480050  0.877226  0.005120 -0.012721 -0.001125 -0.999918    45.408   -12.736    15.793
 
 Setting up LSSR interatomic distance restraints
 Interpreting card: NOTE BUSTER_SIM_RESTRAIN_LSSR ncsautoAB 6.666
        setting up interatomic distance pair restraints with:
        Dmin=         0.00
        Dmax=         5.50
        sigma=        0.20
        Vmax=         3.00
        weight=       6.67
        number of distance pairs defined for this set is      34483
 Have not supplied either '-sim_swap_equiv' or '-sim_swap_equiv_plus' argument 
   so will not try swaping equivalent atoms to improve NCS and/or target agreement.
 
 NCS by LSSR outlier analysis
 -autoncs option automatic NCS prune routine -------- 
 Residues pruned out by gradient condition:
          A|132     A|135     A|139     A|140     A|147     A|148     A|149
          A|150     A|151     A|158     A|169     A|172     A|173     A|175
          A|180     A|181     A|182     A|183     A|184     A|185     A|186
          A|187     A|189     A|216     A|217     A|218     A|219     A|220
          A|221     A|222     A|237     A|253     A|259     A|310     A|319
          A|326     A|328     A|344     A|345     A|346     B|132     B|135
          B|139     B|140     B|147     B|148     B|149     B|150     B|151
          B|158     B|169     B|172     B|173     B|175     B|180     B|181
          B|182     B|183     B|184     B|185     B|186     B|187     B|189
          B|216     B|217     B|218     B|219     B|220     B|221     B|222
          B|237     B|253     B|259     B|310     B|319     B|326     B|328
          B|344     B|345     B|346
  
 Residues pruned out by function/max condition:
          A|149!    A|187!    A|188     A|326!    A|346!   A|ENDA     B|149!
          B|187!    B|188     B|326!    B|346!   B|ENDA 
  
 Total number of residues for which LSSR restraints have been pruned out is       84 out of      698 participating
 Number of LSSR restraints pruned out is      5379 this is   15.6% of the total           34483
  
 -autoncs .Gelly cards suitable for adaptation for manual definition/pruning
           (note auto pruning is done in a slightly different way so exact results may differ slightly)
 -autoncs option .Gelly cards also written to file  auto_gelly_cards.txt
NOTE BUSTER_SET ncsautoXcld = Water    # card added - produced by -autoncs
NOTE BUSTER_SET ncsautoXcld = ncsautoXcld + { A|132 A|135 A|139 A|140 A|147 }    # card added - produced by -autoncs
NOTE BUSTER_SET ncsautoXcld = ncsautoXcld + { A|148 A|149 A|150 A|151 A|158 }    # card added - produced by -autoncs
NOTE BUSTER_SET ncsautoXcld = ncsautoXcld + { A|169 A|172 A|173 A|175 A|180 }    # card added - produced by -autoncs
NOTE BUSTER_SET ncsautoXcld = ncsautoXcld + { A|181 A|182 A|183 A|184 A|185 }    # card added - produced by -autoncs
NOTE BUSTER_SET ncsautoXcld = ncsautoXcld + { A|186 A|187 A|188 A|189 A|216 }    # card added - produced by -autoncs
NOTE BUSTER_SET ncsautoXcld = ncsautoXcld + { A|217 A|218 A|219 A|220 A|221 }    # card added - produced by -autoncs
NOTE BUSTER_SET ncsautoXcld = ncsautoXcld + { A|222 A|237 A|253 A|259 A|310 }    # card added - produced by -autoncs
NOTE BUSTER_SET ncsautoXcld = ncsautoXcld + { A|319 A|326 A|328 A|344 A|345 }    # card added - produced by -autoncs
NOTE BUSTER_SET ncsautoXcld = ncsautoXcld + { A|346 A|ENDA }    # card added - produced by -autoncs
NOTE BUSTER_SET ncsautoABset = Chain_A \ ncsautoXcld    # card added - produced by -autoncs
NOTE BUSTER_SIM_DEFINE ncsautoAB ncsautoABset B    # card added - produced by -autoncs
NOTE BUSTER_SIM_RESTRAIN_LSSR ncsautoAB 6.666    # card added - produced by -autoncs

 LSSR function contribution before pruning     35267.4
                             after pruning      9107.3
 Graph of which residues pruned from LSSR: ncs_pruned.mtv
 Graph of LSSR function outliers ncs_function_outliers.mtv
 Graph of LSSR gradient outliers ncs_gradient_outliers.mtv
 LSSR - analysis of distance difference distribution for NCS
         DeltaD less than 0.5 Angs:  proportion of population= 98.85% average DeltaD of subset     0.012 Angs
 Histogram of LSSR deltaD distribution ncs_histogram.mtv
 
 Interpreting card: NOTE BUSTER_TLS_SET tls1 { A|* }
 Interpreting card: NOTE BUSTER_TLS_SET tls2 { B|* }
 Interpreting card: NOTE BUSTER_TLS_SET tls1 { A|* }
 Interpreting card: NOTE BUSTER_TLS_T   tls1     -1.00387E-01 -1.94646E-02 -1.04862E-01 -2.75119E-02  4.61084E-02  5.22733E-03
 Interpreting card: NOTE BUSTER_TLS_L   tls1      1.43977E+00  1.66678E+00  8.28191E-01  2.91853E-01 -4.93436E-01  3.60273E-01
 Interpreting card: NOTE BUSTER_TLS_S   tls1      2.45347E-01 -2.02592E-01  2.44710E-01 -1.91009E-01 -7.07893E-02 -2.32867E-01 -5.33582E-02 -7.54950E-02
 Interpreting card: NOTE BUSTER_TLS_O   tls1     -2.34016E+01 -8.37990E+01  1.17779E+01
 Interpreting card: NOTE BUSTER_TLS_FIX tls1 ALL
 Interpreting card: NOTE BUSTER_TLS_SET tls2 { B|* }
 Interpreting card: NOTE BUSTER_TLS_T   tls2     -1.34545E-01 -7.70270E-02  4.06613E-02  2.21513E-02  4.79797E-02 -1.95693E-02
 Interpreting card: NOTE BUSTER_TLS_L   tls2      1.12658E+00  1.16836E+00  9.36760E-01  5.85777E-01 -6.91255E-01 -5.92143E-01
 Interpreting card: NOTE BUSTER_TLS_S   tls2     -4.81861E-02  5.31306E-02 -8.29736E-02  4.54205E-02  2.18779E-01  5.84247E-02 -8.79871E-02  4.73445E-02
 Interpreting card: NOTE BUSTER_TLS_O   tls2      2.21707E+01 -7.20650E+01  3.37962E+00
 Interpreting card: NOTE BUSTER_TLS_FIX tls2 ALL


 TLS information. Total number of groups:     2


 Group   Natoms    FixRB   FixTLS   Group Tag    Group Spec
     1     2770      Y        Y     tls1    { A|* }
     2     2770      Y        Y     tls2    { B|* }
 
NOTE BUSTER_TLS_SET tls1 { A|* }
NOTE BUSTER_TLS_T   tls1     -1.00387E-01 -1.94646E-02 -1.04862E-01 -2.75119E-02  4.61084E-02  5.22733E-03
NOTE BUSTER_TLS_L   tls1      1.43977E+00  1.66678E+00  8.28191E-01  2.91853E-01 -4.93436E-01  3.60273E-01
NOTE BUSTER_TLS_S   tls1      2.45347E-01 -2.02592E-01  2.44710E-01 -1.91009E-01 -7.07893E-02 -2.32867E-01 -5.33582E-02 -7.54950E-02
NOTE BUSTER_TLS_O   tls1     -2.34016E+01 -8.37990E+01  1.17779E+01
NOTE BUSTER_TLS_FIX tls1 ALL
 
NOTE BUSTER_TLS_SET tls2 { B|* }
NOTE BUSTER_TLS_T   tls2     -1.34545E-01 -7.70270E-02  4.06613E-02  2.21513E-02  4.79797E-02 -1.95693E-02
NOTE BUSTER_TLS_L   tls2      1.12658E+00  1.16836E+00  9.36760E-01  5.85777E-01 -6.91255E-01 -5.92143E-01
NOTE BUSTER_TLS_S   tls2     -4.81861E-02  5.31306E-02 -8.29736E-02  4.54205E-02  2.18779E-01  5.84247E-02 -8.79871E-02  4.73445E-02
NOTE BUSTER_TLS_O   tls2      2.21707E+01 -7.20650E+01  3.37962E+00
NOTE BUSTER_TLS_FIX tls2 ALL


 TLS: mmCIF group specification begin


#
loop_
_pdbx_refine_tls_group.id
_pdbx_refine_tls_group.refine_tls_id
_pdbx_refine_tls_group.beg_PDB_ins_code
_pdbx_refine_tls_group.beg_auth_asym_id
_pdbx_refine_tls_group.beg_auth_seq_id
_pdbx_refine_tls_group.end_PDB_ins_code
_pdbx_refine_tls_group.end_auth_asym_id
_pdbx_refine_tls_group.end_auth_seq_id
_pdbx_refine_tls_group.selection
_pdbx_refine_tls_group.selection_details
   1   1 . A   -1 . A  346 . '{ A|* }'
   2   1 . A  401 . A  401 . '{ A|* }'
   3   2 . B   -1 . B  346 . '{ B|* }'
   4   2 . B  401 . B  401 . '{ B|* }'
#


 TLS: mmCIF group specification end


 Interpreting card: NOTE BUSTER_ANISO_SET Empty
 Interpreting card: NOTE BUSTER_ANISO_WEIGHT SCA  16.0
 Interpreting card: NOTE BUSTER_ANISO_WEIGHT SPH   0.6    0.6
 Interpreting card: NOTE BUSTER_ANISO_WEIGHT SIM   7.8    4.8    2.6    0
 Interpreting card: NOTE BUSTER_ANISO_WEIGHT RIG  21.5   21.5    3.7    0


 After TLS and ANISO: Number of atoms with fixed/combined xyz =     0
 After TLS and ANISO: Number of atoms with fixed/combined B   =     0
 After TLS and ANISO: Number of atoms with fixed/combined OCC =  6506
 After TLS and ANISO: Number of atoms with fixed/combined BIJ =  6506
 After TLS and ANISO: Number of atoms in a TLS group          =  5540
                                     cf total number of atoms =  6506
 

 ############### Setup state vector

 No free_vect_ulim_xyz argument found: using default of  100.000
 No free_vect_llim_xyz argument found: using default of -100.000
 Number of parameters added for TLS description                 =       0
 Number of parameters to describe molecular conformation        =   26024

 ############### Setup free vector

 Number of free parameters to be adjusted in refinement   =   26024

 ############### Setting up own geometry


 No command line argument '-tnt_fn' so will use gelly to do geometry function calculations

 ============= Call TNT functions


 ============= Get TNT symmetry information

 
      Getting symmetry operators from TNT. 
          gelly will classify symmetry using pdb-like convention: 
               SYMOP   SYMMETRY
              NNNMMM   OPERATOR
                1555   X,Y,Z
                2555   -Y,X-Y,Z
                3555   -X+Y,-X,Z
                4555   -X,-Y,1/2+Z
                5555   Y,-X+Y,1/2+Z
                6555   X-Y,X,1/2+Z
          where NNN -> operator number and MMM -> translation vector
 

 ============= Own Gelly geometry/restraints setup


 ----------- Identify bond-types


    Setting up GEOMBONDREC_TYPE_HASH* to be able to map a given bond type (e.g.
    "single") to an internal integer (GBOND_TYPE=1) etc.

    Using default  GEOMBONDREC_TYPE_HASH_STR='SING* 1 DOUB* 2 TRIP* 3 AROM* 4 DELO* 4 METAL* 5':
    to override default use a NOTE BUSTER_GEOMBONDREC_TYPE_HASH_STR gelly card.

 ----------- Classify torsion into peptide omega and other


 ----------- Setup of non-bonded terms

 Ideal-distance contact term setup:
    Restraint function constants (normally these values are from NOTE BUSTER_IDEAL_CONTACT_* cards)
    Sigma for general contacts      0.200 Angs
    Sigma for 1-4 contacts          0.100 Angs (contacts across a torsion)
    Sigma for 1-5 contacts          0.150 Angs
    Sigma for 1-N contacts          0.200 Angs
    Sigma for HBOND pairs           0.100 Angs (contacts between HB_types A and D or B's)
    Contact distance increment for each atom in HBOND          -0.150 Angs 
    Contact distance increment for H-bond acceptor              0.100 Angs 
    Contact distance increment for 1-4 with an O atom          -0.050 Angs 
    Contact distance increment for 1-4 with an N atom          -0.180 Angs 
    Contact distance increment for 1-4 with an C atom          -0.370 Angs 
    Contact distance increment for 1-4 with an S atom          -0.150 Angs 
    Contact distance increment for 1-4 with an P atom          -0.150 Angs 
    Contact distance increment for 1-4 with any other atom     -0.150 Angs 
    All atoms matched for CCP4 atom type.
    Number of CCP4 atom types used is         25 (H-bound and not H-bound counted separately)

 Setting up short/ideal-distance contacts
   Using gelly special position handling. (For TNT instead rerun with argument '-special_tnt')

   No command line argument '-special_all' specified. We will auto-generate
   EXCLUDE cards only for atom-atom contacts across symmetry (i.e. on special
   position) when this involves a contact within a residue to itself.

   Have set up non-bonded exclusions - 1-3 contacts
   Atom A|27:CA         makes the most 1-3 contacts:  6

   Have set up non-bonded exclusions - 1-4 contacts
   Atom A|27:N          makes the most 1-4 contacts:  7

   Have set up non-bonded exclusions - 1-5 contacts
   Atom A|28:C.A        makes the most 1-5 contacts: 10

 ############################################################################################################
 ###################################################### setting up contact terms
 ############################################################################################################


 ============= Call Gelly versions of TNT functions

 WARNING: 1-4 contact without 1-(2,3)-4 data for A|401:O2(CIT)        and A|401:C3(CIT)        X-X
 WARNING: 1-4 contact without 1-(2,3)-4 data for A|401:C2(CIT)        and A|401:O6(CIT)        X-X
 WARNING: 1-4 contact without 1-(2,3)-4 data for A|401:O7(CIT)        and A|401:O5(CIT)        X-X
 WARNING: 1-4 contact without 1-(2,3)-4 data for A|401:C4(CIT)        and A|401:O6(CIT)        X-X
 WARNING: 1-4 contact without 1-(2,3)-4 data for B|401:C01(LIG)       and B|401:N05(LIG)       X-X
 WARNING: 1-4 contact without 1-(2,3)-4 data for B|401:C04(LIG)       and B|401:C13(LIG)       X-X
 WARNING: 1-4 contact without 1-(2,3)-4 data for B|401:C09(LIG)       and B|401:C12(LIG)       X-X
 WARNING: 1-4 contact without 1-(2,3)-4 data for B|401:C10(LIG)       and B|401:C13(LIG)       X-X

       TERM      weight      number      TNT_fn    Gelly_fn        diff
       BOND       2.000        5655    1727.303    1727.303       0.000
       ANGL       2.000        7674    5391.091    5391.091       0.000
       IMPR       0.000           0       0.000       0.000       0.000
       TORS       2.000        1968    3112.251    3112.251      -0.000
       TRIG       2.000           0       0.000       0.000       0.000
       PLAN       5.000         963    2560.280    2560.280       0.000
       CONT       5.000           0     117.614       0.000     117.614
       BCOR      10.000        5630    6959.511    6378.797     580.714


 ============= Call Gelly geometry functions

   Switching torsion functional form to sinusoidal rather than split harmonic functional.
      if you want this not to happen then rerun with '-torsharm'
      Weight for sinusoidal torsion=     2.000
   BCOR function/gradient will be split between H and non-H atoms (as for any atom pair). Use '-bcorrel_all_in_hydrogens' to change this.

 ----------- Fix B-correlation restraints

 
 Checking for bonds that do not have a bcorrel restraint defined
 We will wipe all existing BCORrels if OCC=0.0 atom(s) is/are involved. This can be' switched off via the '-bcorrel_nowipe_zeroocc' flag.
 We will wipe all existing BCORrels if only non-hydrogen atom(s) with fixed B(iso) are involved. This can be switched off via the '-bcorrel_nowipe_fixb_nonhydrogens' flag.
 We will keep all existing BCORrels if hydrogen atom(s) with fixed B(iso) is/are involved. This can be switched off via the '-bcorrel_wipe_fixb_hydrogens' flag.
 No -bcorrel_missing_sigma found. So will set sigma to     2.5 Ang**2 for any bond with no existing BCORrel
 No -bcorrel_missing_sigma_hydrogens found. So will set sigma to     1.0 Ang**2 for any bond with no existing BCORrel involving hydrogens

 No active BCORels found involving OCC=0.0 atom(s)


 No active BCORels found involving fixed-B (only) non-hydrogen atom(s)

    add a default BCOR for bond A|401:C1(CIT) A|401:C2(CIT)
    add a default BCOR for bond A|401:C1(CIT) A|401:O1(CIT)
    add a default BCOR for bond A|401:C1(CIT) A|401:O2(CIT)
    add a default BCOR for bond A|401:C2(CIT) A|401:C3(CIT)
    add a default BCOR for bond A|401:C3(CIT) A|401:C4(CIT)
    add a default BCOR for bond A|401:C3(CIT) A|401:C6(CIT)
    add a default BCOR for bond A|401:C3(CIT) A|401:O7(CIT)
    add a default BCOR for bond A|401:C4(CIT) A|401:C5(CIT)
    add a default BCOR for bond A|401:C5(CIT) A|401:O3(CIT)
    add a default BCOR for bond A|401:C5(CIT) A|401:O4(CIT)
    add a default BCOR for bond A|401:C6(CIT) A|401:O5(CIT)
    add a default BCOR for bond A|401:C6(CIT) A|401:O6(CIT)
    add a default BCOR for bond B|401:C01(LIG) B|401:C02(LIG)
    add a default BCOR for bond B|401:C02(LIG) B|401:C03(LIG)
    add a default BCOR for bond B|401:C02(LIG) B|401:C04(LIG)
    add a default BCOR for bond B|401:C04(LIG) B|401:N05(LIG)
    add a default BCOR for bond B|401:C04(LIG) B|401:C06(LIG)
    add a default BCOR for bond B|401:C06(LIG) B|401:O07(LIG)
    add a default BCOR for bond B|401:C06(LIG) B|401:N08(LIG)
    add a default BCOR for bond B|401:N08(LIG) B|401:C09(LIG)
    add a default BCOR for bond B|401:N08(LIG) B|401:C13(LIG)
    add a default BCOR for bond B|401:C09(LIG) B|401:C10(LIG)
    add a default BCOR for bond B|401:C10(LIG) B|401:O11(LIG)
    add a default BCOR for bond B|401:O11(LIG) B|401:C12(LIG)
    add a default BCOR for bond B|401:C12(LIG) B|401:C13(LIG)

 Have added a total of        25 BCORrels for bonds which do not already have a BCORrel


 No bonds (for potential BCORrel addition) were skipped due to both atoms having constant/fixed B(iso)


 ----------- Deactivate hydrogen-free planes if hydrogen atoms present

 A total of       0 planes have been deactivated because they are duplicated hydrogen-free and with-hydrogen forms.

 ----------- Handle peptide omega angle(s)

      Torsion angles split into peptide omega and other
         Number of peptide omega angles is       698
         Number of other torsion is             1270
      Peptide omega angles are monitored rather than directly restrained (PLANE used instead).

 ----------- Fix cis-PRO

 Now apply the fix to get correct restraints for cis PRO residues
   Have got      11 bonds records and      26 angles
   from GEOMETRY cards for the special PROC residue
   Weight for ideal-distance contact term=     4.000

 ----------- Prepare SSBOND/LINK for output PDB file


 ----------- Prepare for QM


 ----------- Prepare symmetry in utility distance restraints


 ############### Setting up Rhofit contacts (if needed)

 No ligand-(fixed protein) contact term will be used

 ############### Working out initial function and gradient


   MaxLik Scaling Cycle 0002
 
 Initial functional value = 4.426399E+05 rms gradient=      21.0638
 X-ray contribution to function value = 4.039127E+05
 Geometry contribution (inc ncs)      =      38727.2
 Geometry function summary
     term                           number     weight       rms        GooF      function     fn/numb
     ================================================================================================
     BOND bond lengths (angs)       5655       2.000      0.0078       0.391      1727.30       0.305
     ANGL bond angles (degs)        7674       2.000        0.99       0.593      5391.09       0.703
     TORS split-harmonic tors       1270       0.000       17.60       1.107         0.00       0.000
     SINTOR sinusoidal tors         1270       2.000       17.60                  2374.47       1.870
     PLAN general plane (angs)       963       5.000      0.0146       0.729      2560.28       2.659
     BCORrelations (angs^2)         5655      10.000       0.941       0.336      6379.71       1.128
     CONT (bad contacts)               0       5.000                                 0.00
     IDEAL(ideal-dist contact)      5386       4.000                             11134.13
     CHIRAL (gelly semiharmon)       740       5.000                                52.87
     SIM similarity restraint for NCS and/or TARGET                               9107.33
     OCCSUM restraint on sum of occupanies                                           0.00       as none defined
     DISTAN utility restraints interatomic distance                                  0.00       as none defined
     UTILANGLE utility angle restraints                                              0.00       as none defined
     UTILTOR utility restraints on torsion angles                                    0.00       as none defined
     Peptide omega torsion angles                           2.96 degs - monitor only

 Weighted rms      0.0078         0.995  (temporary rms bond and angle for BUSTER to pick up!)
 Weighted intra-residual rms(bond) for StdProtein (excluding H)      0.0076
 Weighted intra-residual rms(bond) for Buffer     (excluding H)      0.0030

 ############### Running initial check


 Initial Geometry Sanity Check

 Check for duplicate bonds: no duplicates found
 
 Check for duplicate or missing bond angles: all are OK
 Check for bond between an atom and itself not involving symmetry:  all are OK
   There are no bad contacts where Delta/sigma is greater than     6.5
   Worst contact dist=   2.685 std=   3.502 sigma=   0.222 abs(Delta/sigma)=    3.681 ats B|104:SD (MET) W|390:O (HOH)
   It is likely that the geometry libraries, sequence info and links match the input molecule
 GEOMETRY CHECK OVERALL:  PASS


 ############### Creating geometry/screen report


 ############### Option "tao" - run Tao optimizer

 TAO: Toolkit for Advanced Optimization. Reference: 
     Munson, T., Sarich, J., Wild, S., Benson, S., McInnes, L.C. (2018)
     "TAO User Manual (Revision 3.9)", Mathematics and Computer Science
     Division, Argonne National Laboratory
     ANL/MCS-TM-322, http://www.mcs.anl.gov/petsc/
 
 Have found -keeppdb flag so will write out a .pdb file every 1000000 its during opt
 Will apply TAO optimization 
 Maximum number of iterations      100
 Convergence test on Grms       4.0000 (-glim)
 Limit on rms displacement to original position (rmsD2init): NONE test inactive (-dlim)

    analysing argument no 0 : -autoncs with j=0
    analysing argument no 1 : -hydrogens_input_occ2zero with j=0
    analysing argument no 2 : -hydrogens_output_occ2heavy with j=0
    analysing argument no 3 : -screen with j=0
    analysing argument no 4 : 100 with j=0
    analysing argument no 5 : -screen_sigma with j=0
    analysing argument no 6 : 3.0 with j=0
    analysing argument no 7 : -keeppdb with j=0
    analysing argument no 8 : 1000000 with j=0
    analysing argument no 9 : -taomonitor_verbose with j=0
    found argument starting with "-tao" : -taomonitor_verbose
    analysing argument no 10 : 2 with j=1
    found value after -tao/-options_/-viewer_ argument : "2"
 PETSC/TAO: setting option "-taomonitor_verbose" = "2"


   MaxLik Scaling Cycle 0003
   Iters     Tot_Funct    Xray_Funct    Geom_Funct          Grms     rmsD2init     maxD2init             R         Rfree           LLG       LLGfree     rms(bond)    rms(angle)       Opttag_H
   Iters             K   B_IMPF_FRAG        K_MISS   B_TEMP_MISS   K_IMPF_MISS   B_IMPF_MISS        K_SOLV   B_TEMP_SOLV   K_IMPF_SOLV   B_IMPF_SOLV        B_TEMP     B_TEMP_11     B_TEMP_12     B_TEMP_22     B_TEMP_13     B_TEMP_23     B_TEMP_33      K_WILSON      B_WILSON      FGCYCLES       Scatag_H
   Iters          BOND         ANGLE      IMPROPER       TORSION    SINTORSION      TRIGONAL        PLANAR  BCORRELATION       CONTACT     IDEALDIST        CHIRAL    SIMILARITY       OCCSSUM  UTILDISTANCE     UTILANGLE   UTILTORSION        RHOFIT        ADP-01        ADP-02        ADP-03        ADP-04        ADP-05        ADP-06        ADP-07        ADP-08        ADP-09        ADP-10      RHOFITPC            QM       Funtag_H
       0  4.426399E+05  4.039127E+05       38727.2       21.0638  0.000000E+00  0.000000E+00      0.184224      0.226227  0.000000E+00  0.000000E+00    0.00784184      0.994540       Opttag_D
       0       76.6430       1.05922       1.00000  0.000000E+00       1.00000  0.000000E+00      0.929546       69.9407       1.00000       34.5853  0.000000E+00       8.94664  0.000000E+00       8.94664  0.000000E+00  0.000000E+00      -17.8933       78.9652       3.74714        2       Scatag_D
       0       1727.30       5391.09  0.000000E+00  0.000000E+00       2374.47  0.000000E+00       2560.28       6379.71  0.000000E+00       11134.1       52.8700       9107.33 -1.000000E+10 -1.000000E+10 -1.000000E+10 -1.000000E+10 -1.000000E+10  0.000000E+00  0.000000E+00  0.000000E+00  0.000000E+00  0.000000E+00  0.000000E+00  0.000000E+00  0.000000E+00  0.000000E+00  0.000000E+00  0.000000E+00  0.000000E+00       Funtag_D
   Geometry Restraint Deviations SCREEN Information

 

 WARNING: have displacement of 20.83 Angs from initial (> 15.00 Angs)
          for variable number= 7114 (type Y        ; returned function value as   3.121966E+010 )
          Involving atom A|225:CG

 An atom has moved more than     1.50 A from the initial/last
 position - if this was an accepted trial point, probably need to update the
 non-bonded contact list.


   MaxLik Scaling Cycle 0004

   MaxLik Scaling Cycle 0005

   MaxLik Scaling Cycle 0006

   Wilson plot

   Statistics in resolution bins after refinement cycle 0001
```


|  |  |
| --- | --- |
|  | - Rxpct factors plot- Rxpct factors table- <Log-Lik Gain>- Ln Sigma A- Correlation Coefficients |


```
   Iters     Tot_Funct    Xray_Funct    Geom_Funct          Grms     rmsD2init     maxD2init             R         Rfree           LLG       LLGfree     rms(bond)    rms(angle)
   Iters             K   B_IMPF_FRAG        K_MISS   B_TEMP_MISS   K_IMPF_MISS   B_IMPF_MISS        K_SOLV   B_TEMP_SOLV   K_IMPF_SOLV   B_IMPF_SOLV        B_TEMP     B_TEMP_11     B_TEMP_12     B_TEMP_22     B_TEMP_13     B_TEMP_23     B_TEMP_33      K_WILSON      B_WILSON      FGCYCLES
   Iters          BOND         ANGLE      IMPROPER       TORSION    SINTORSION      TRIGONAL        PLANAR  BCORRELATION       CONTACT     IDEALDIST        CHIRAL    SIMILARITY       OCCSSUM  UTILDISTANCE     UTILANGLE   UTILTORSION        RHOFIT        ADP-01        ADP-02        ADP-03        ADP-04        ADP-05        ADP-06        ADP-07        ADP-08        ADP-09        ADP-10      RHOFITPC            QM
       1  4.425823E+05  4.038579E+05       38724.5       13.7654  2.527089E-04    0.00334513      0.184190      0.226225  1.475176E-04 -6.306815E-05    0.00784834      0.994326       Opttag_D
       1       76.6434       1.05793       1.00000  0.000000E+00       1.00000  0.000000E+00      0.929543       69.9406       1.00000       34.5732  0.000000E+00       8.94727  0.000000E+00       8.94727  0.000000E+00  0.000000E+00      -17.8945       78.9652       3.74714        3       Scatag_D
       1       1730.17       5388.77  0.000000E+00  0.000000E+00       2374.52  0.000000E+00       2560.94       6380.70  0.000000E+00       11134.4       52.8545       9102.13 -1.000000E+10 -1.000000E+10 -1.000000E+10 -1.000000E+10 -1.000000E+10  0.000000E+00  0.000000E+00  0.000000E+00  0.000000E+00  0.000000E+00  0.000000E+00  0.000000E+00  0.000000E+00  0.000000E+00  0.000000E+00  0.000000E+00  0.000000E+00       Funtag_D
   Geometry Restraint Deviations SCREEN Information

 

   MaxLik Scaling Cycle 0007

   Wilson plot

   Statistics in resolution bins after refinement cycle 0002
```


|  |  |
| --- | --- |
|  | - Rxpct factors plot- Rxpct factors table- <Log-Lik Gain>- Ln Sigma A- Correlation Coefficients |


```
   Iters     Tot_Funct    Xray_Funct    Geom_Funct          Grms     rmsD2init     maxD2init             R         Rfree           LLG       LLGfree     rms(bond)    rms(angle)
   Iters             K   B_IMPF_FRAG        K_MISS   B_TEMP_MISS   K_IMPF_MISS   B_IMPF_MISS        K_SOLV   B_TEMP_SOLV   K_IMPF_SOLV   B_IMPF_SOLV        B_TEMP     B_TEMP_11     B_TEMP_12     B_TEMP_22     B_TEMP_13     B_TEMP_23     B_TEMP_33      K_WILSON      B_WILSON      FGCYCLES
   Iters          BOND         ANGLE      IMPROPER       TORSION    SINTORSION      TRIGONAL        PLANAR  BCORRELATION       CONTACT     IDEALDIST        CHIRAL    SIMILARITY       OCCSSUM  UTILDISTANCE     UTILANGLE   UTILTORSION        RHOFIT        ADP-01        ADP-02        ADP-03        ADP-04        ADP-05        ADP-06        ADP-07        ADP-08        ADP-09        ADP-10      RHOFITPC            QM
       2  4.424840E+05  4.036582E+05       38825.8       13.7974    0.00113414    0.00825107      0.184061      0.226207  6.830298E-04 -3.022392E-04    0.00791659      0.995389       Opttag_D
       2       76.6461       1.05371       1.00000  0.000000E+00       1.00000  0.000000E+00      0.929502       69.9655       1.00000       34.5156  0.000000E+00       8.94853  0.000000E+00       8.94853  0.000000E+00  0.000000E+00      -17.8971       78.9652       3.74714        1       Scatag_D
       2       1760.39       5400.30  0.000000E+00  0.000000E+00       2374.64  0.000000E+00       2565.34       6445.47  0.000000E+00       11137.1       53.2497       9089.31 -1.000000E+10 -1.000000E+10 -1.000000E+10 -1.000000E+10 -1.000000E+10  0.000000E+00  0.000000E+00  0.000000E+00  0.000000E+00  0.000000E+00  0.000000E+00  0.000000E+00  0.000000E+00  0.000000E+00  0.000000E+00  0.000000E+00  0.000000E+00       Funtag_D
   Geometry Restraint Deviations SCREEN Information

 
       3  4.424109E+05  4.034065E+05       39004.3       18.6715    0.00244511     0.0200802      0.183895      0.226176    0.00135802 -5.003889E-04    0.00802737      0.997980       Opttag_D
       3       76.6517       1.04902       1.00000  0.000000E+00       1.00000  0.000000E+00      0.929421       70.0282       1.00000       34.4290  0.000000E+00       8.94930  0.000000E+00       8.94930  0.000000E+00  0.000000E+00      -17.8986       78.9652       3.74714        1       Scatag_D
       3       1810.00       5428.45  0.000000E+00  0.000000E+00       2375.02  0.000000E+00       2575.16       6526.00  0.000000E+00       11145.8       53.6338       9090.26 -1.000000E+10 -1.000000E+10 -1.000000E+10 -1.000000E+10 -1.000000E+10  0.000000E+00  0.000000E+00  0.000000E+00  0.000000E+00  0.000000E+00  0.000000E+00  0.000000E+00  0.000000E+00  0.000000E+00  0.000000E+00  0.000000E+00  0.000000E+00       Funtag_D
       4  4.423561E+05  4.032617E+05       39094.4       17.9662    0.00330379     0.0288971      0.183798      0.226165    0.00174648 -6.336633E-04    0.00807829      0.999371       Opttag_D
       4       76.6563       1.04659       1.00000  0.000000E+00       1.00000  0.000000E+00      0.929362       70.0773       1.00000       34.3743  0.000000E+00       8.94900  0.000000E+00       8.94900  0.000000E+00  0.000000E+00      -17.8980       78.9652       3.74714        1       Scatag_D
       4       1833.04       5443.59  0.000000E+00  0.000000E+00       2375.49  0.000000E+00       2582.70       6556.17  0.000000E+00       11154.3       53.6272       9095.54 -1.000000E+10 -1.000000E+10 -1.000000E+10 -1.000000E+10 -1.000000E+10  0.000000E+00  0.000000E+00  0.000000E+00  0.000000E+00  0.000000E+00  0.000000E+00  0.000000E+00  0.000000E+00  0.000000E+00  0.000000E+00  0.000000E+00  0.000000E+00       Funtag_D
       5  4.423043E+05  4.031762E+05       39128.1       14.9096    0.00393637     0.0361386      0.183739      0.226162    0.00197572 -7.089294E-04    0.00808863      0.999703       Opttag_D
       5       76.6602       1.04546       1.00000  0.000000E+00       1.00000  0.000000E+00      0.929311       70.1220       1.00000       34.3356  0.000000E+00       8.94820  0.000000E+00       8.94820  0.000000E+00  0.000000E+00      -17.8964       78.9652       3.74714        1       Scatag_D
       5       1837.73       5447.20  0.000000E+00  0.000000E+00       2375.91  0.000000E+00       2588.30       6566.83  0.000000E+00       11161.6       53.5357       9097.00 -1.000000E+10 -1.000000E+10 -1.000000E+10 -1.000000E+10 -1.000000E+10  0.000000E+00  0.000000E+00  0.000000E+00  0.000000E+00  0.000000E+00  0.000000E+00  0.000000E+00  0.000000E+00  0.000000E+00  0.000000E+00  0.000000E+00  0.000000E+00       Funtag_D
       6  4.422258E+05  4.030004E+05       39225.4       16.9283    0.00546450     0.0534454      0.183616      0.226161    0.00244739 -8.740993E-04    0.00811732       1.00181       Opttag_D
       6       76.6696       1.04341       1.00000  0.000000E+00       1.00000  0.000000E+00      0.929190       70.2270       1.00000       34.2509  0.000000E+00       8.94610  0.000000E+00       8.94610  0.000000E+00  0.000000E+00      -17.8922       78.9652       3.74714        1       Scatag_D
       6       1850.79       5470.16  0.000000E+00  0.000000E+00       2376.92  0.000000E+00       2601.24       6590.20  0.000000E+00       11180.8       53.7331       9101.59 -1.000000E+10 -1.000000E+10 -1.000000E+10 -1.000000E+10 -1.000000E+10  0.000000E+00  0.000000E+00  0.000000E+00  0.000000E+00  0.000000E+00  0.000000E+00  0.000000E+00  0.000000E+00  0.000000E+00  0.000000E+00  0.000000E+00  0.000000E+00       Funtag_D
       7  4.421780E+05  4.027943E+05       39383.7       29.0189    0.00745159     0.0756698      0.183471      0.226174    0.00299999   -0.00110568    0.00815591       1.00652       Opttag_D
       7       76.6813       1.04096       1.00000  0.000000E+00       1.00000  0.000000E+00      0.929043       70.3478       1.00000       34.1538  0.000000E+00       8.94395  0.000000E+00       8.94395  0.000000E+00  0.000000E+00      -17.8879       78.9652       3.74714        1       Scatag_D
       7       1868.43       5521.71  0.000000E+00  0.000000E+00       2378.36  0.000000E+00       2616.27       6628.20  0.000000E+00       11208.9       54.0709       9107.73 -1.000000E+10 -1.000000E+10 -1.000000E+10 -1.000000E+10 -1.000000E+10  0.000000E+00  0.000000E+00  0.000000E+00  0.000000E+00  0.000000E+00  0.000000E+00  0.000000E+00  0.000000E+00  0.000000E+00  0.000000E+00  0.000000E+00  0.000000E+00       Funtag_D
       8  4.421069E+05  4.027408E+05       39366.1       14.9166    0.00798634     0.0818467      0.183434      0.226181    0.00314361   -0.00117363    0.00814133       1.00454       Opttag_D
       8       76.6842       1.04027       1.00000  0.000000E+00       1.00000  0.000000E+00      0.929004       70.3770       1.00000       34.1303  0.000000E+00       8.94373  0.000000E+00       8.94373  0.000000E+00  0.000000E+00      -17.8875       78.9652       3.74714        1       Scatag_D
       8       1861.75       5500.04  0.000000E+00  0.000000E+00       2378.78  0.000000E+00       2618.41       6633.12  0.000000E+00       11216.8       54.0710       9103.18 -1.000000E+10 -1.000000E+10 -1.000000E+10 -1.000000E+10 -1.000000E+10  0.000000E+00  0.000000E+00  0.000000E+00  0.000000E+00  0.000000E+00  0.000000E+00  0.000000E+00  0.000000E+00  0.000000E+00  0.000000E+00  0.000000E+00  0.000000E+00       Funtag_D
       9  4.420393E+05  4.026366E+05       39402.7       12.9586    0.00908929     0.0940853      0.183360      0.226192    0.00342295   -0.00129633    0.00813268       1.00533       Opttag_D
       9       76.6903       1.03895       1.00000  0.000000E+00       1.00000  0.000000E+00      0.928925       70.4353       1.00000       34.0850  0.000000E+00       8.94329  0.000000E+00       8.94329  0.000000E+00  0.000000E+00      -17.8866       78.9652       3.74714        1       Scatag_D
       9       1857.80       5508.72  0.000000E+00  0.000000E+00       2379.64  0.000000E+00       2622.22       6650.40  0.000000E+00       11234.0       54.2955       9095.60 -1.000000E+10 -1.000000E+10 -1.000000E+10 -1.000000E+10 -1.000000E+10  0.000000E+00  0.000000E+00  0.000000E+00  0.000000E+00  0.000000E+00  0.000000E+00  0.000000E+00  0.000000E+00  0.000000E+00  0.000000E+00  0.000000E+00  0.000000E+00       Funtag_D
      10  4.419821E+05  4.025139E+05       39468.1       15.4379     0.0105752      0.109627      0.183271      0.226199    0.00375210   -0.00142581    0.00813042       1.00701       Opttag_D
      10       76.6989       1.03763       1.00000  0.000000E+00       1.00000  0.000000E+00      0.928819       70.5163       1.00000       34.0275  0.000000E+00       8.94225  0.000000E+00       8.94225  0.000000E+00  0.000000E+00      -17.8845       78.9652       3.74714        1       Scatag_D
      10       1856.77       5527.18  0.000000E+00  0.000000E+00       2380.82  0.000000E+00       2627.85       6670.98  0.000000E+00       11258.0       54.4925       9092.10 -1.000000E+10 -1.000000E+10 -1.000000E+10 -1.000000E+10 -1.000000E+10  0.000000E+00  0.000000E+00  0.000000E+00  0.000000E+00  0.000000E+00  0.000000E+00  0.000000E+00  0.000000E+00  0.000000E+00  0.000000E+00  0.000000E+00  0.000000E+00       Funtag_D
      11  4.419383E+05  4.022935E+05       39644.8       29.4384     0.0139262      0.141366      0.183107      0.226217    0.00434334   -0.00162432    0.00812554       1.01196       Opttag_D
      11       76.7188       1.03590       1.00000  0.000000E+00       1.00000  0.000000E+00      0.928576       70.6994       1.00000       33.9146  0.000000E+00       8.93969  0.000000E+00       8.93969  0.000000E+00  0.000000E+00      -17.8794       78.9652       3.74714        1       Scatag_D
      11       1854.54       5581.64  0.000000E+00  0.000000E+00       2383.58  0.000000E+00       2638.33       6723.54  0.000000E+00       11314.3       54.7620       9094.08 -1.000000E+10 -1.000000E+10 -1.000000E+10 -1.000000E+10 -1.000000E+10  0.000000E+00  0.000000E+00  0.000000E+00  0.000000E+00  0.000000E+00  0.000000E+00  0.000000E+00  0.000000E+00  0.000000E+00  0.000000E+00  0.000000E+00  0.000000E+00       Funtag_D
      12  4.418597E+05  4.022695E+05       39590.2       11.9855     0.0144433      0.144926      0.183087      0.226219    0.00440758   -0.00164944    0.00810086       1.00968       Opttag_D
      12       76.7222       1.03589       1.00000  0.000000E+00       1.00000  0.000000E+00      0.928539       70.7298       1.00000       33.8987  0.000000E+00       8.93886  0.000000E+00       8.93886  0.000000E+00  0.000000E+00      -17.8777       78.9652       3.74714        1       Scatag_D
      12       1843.29       5556.45  0.000000E+00  0.000000E+00       2383.93  0.000000E+00       2638.62       6698.69  0.000000E+00       11322.4       54.5952       9092.21 -1.000000E+10 -1.000000E+10 -1.000000E+10 -1.000000E+10 -1.000000E+10  0.000000E+00  0.000000E+00  0.000000E+00  0.000000E+00  0.000000E+00  0.000000E+00  0.000000E+00  0.000000E+00  0.000000E+00  0.000000E+00  0.000000E+00  0.000000E+00       Funtag_D
      13  4.418274E+05  4.022739E+05       39553.5       9.67946     0.0145713      0.144012      0.183090      0.226214    0.00439579   -0.00162269    0.00808221       1.00913       Opttag_D
      13       76.7232       1.03619       1.00000  0.000000E+00       1.00000  0.000000E+00      0.928526       70.7426       1.00000       33.8957  0.000000E+00       8.93844  0.000000E+00       8.93844  0.000000E+00  0.000000E+00      -17.8769       78.9652       3.74714        1       Scatag_D
      13       1834.82       5550.45  0.000000E+00  0.000000E+00       2384.07  0.000000E+00       2636.43       6681.87  0.000000E+00       11323.5       54.4757       9087.88 -1.000000E+10 -1.000000E+10 -1.000000E+10 -1.000000E+10 -1.000000E+10  0.000000E+00  0.000000E+00  0.000000E+00  0.000000E+00  0.000000E+00  0.000000E+00  0.000000E+00  0.000000E+00  0.000000E+00  0.000000E+00  0.000000E+00  0.000000E+00       Funtag_D
      14  4.417792E+05  4.022351E+05       39544.1       11.6280     0.0156016      0.147852      0.183058      0.226215    0.00449975   -0.00163209    0.00806353       1.00940       Opttag_D
      14       76.7297       1.03636       1.00000  0.000000E+00       1.00000  0.000000E+00      0.928451       70.8044       1.00000       33.8671  0.000000E+00       8.93707  0.000000E+00       8.93707  0.000000E+00  0.000000E+00      -17.8741       78.9652       3.74714        1       Scatag_D
      14       1826.35       5553.39  0.000000E+00  0.000000E+00       2384.98  0.000000E+00       2635.27       6664.34  0.000000E+00       11338.8       54.6668       9086.30 -1.000000E+10 -1.000000E+10 -1.000000E+10 -1.000000E+10 -1.000000E+10  0.000000E+00  0.000000E+00  0.000000E+00  0.000000E+00  0.000000E+00  0.000000E+00  0.000000E+00  0.000000E+00  0.000000E+00  0.000000E+00  0.000000E+00  0.000000E+00       Funtag_D
      15  4.417445E+05  4.021250E+05       39619.5       24.7214     0.0178071      0.173315      0.182975      0.226222    0.00479518   -0.00173537    0.00806543       1.01146       Opttag_D
      15       76.7426       1.03565       1.00000  0.000000E+00       1.00000  0.000000E+00      0.928312       70.9117       1.00000       33.8088  0.000000E+00       8.93515  0.000000E+00       8.93515  0.000000E+00  0.000000E+00      -17.8703       78.9652       3.74714        1       Scatag_D
      15       1827.21       5576.04  0.000000E+00  0.000000E+00       2387.30  0.000000E+00       2636.36       6674.20  0.000000E+00       11371.8       55.2948       9091.28 -1.000000E+10 -1.000000E+10 -1.000000E+10 -1.000000E+10 -1.000000E+10  0.000000E+00  0.000000E+00  0.000000E+00  0.000000E+00  0.000000E+00  0.000000E+00  0.000000E+00  0.000000E+00  0.000000E+00  0.000000E+00  0.000000E+00  0.000000E+00       Funtag_D
      16  4.416886E+05  4.020699E+05       39618.7       11.6153     0.0188956      0.187568      0.182936      0.226232    0.00494301   -0.00180557    0.00806102       1.01110       Opttag_D
      16       76.7489       1.03527       1.00000  0.000000E+00       1.00000  0.000000E+00      0.928243       70.9626       1.00000       33.7815  0.000000E+00       8.93417  0.000000E+00       8.93417  0.000000E+00  0.000000E+00      -17.8683       78.9652       3.74714        1       Scatag_D
      16       1825.21       5572.15  0.000000E+00  0.000000E+00       2388.24  0.000000E+00       2638.34       6654.34  0.000000E+00       11388.8       55.5594       9096.06 -1.000000E+10 -1.000000E+10 -1.000000E+10 -1.000000E+10 -1.000000E+10  0.000000E+00  0.000000E+00  0.000000E+00  0.000000E+00  0.000000E+00  0.000000E+00  0.000000E+00  0.000000E+00  0.000000E+00  0.000000E+00  0.000000E+00  0.000000E+00       Funtag_D
      17  4.416562E+05  4.020054E+05       39650.9       8.42968     0.0198663      0.200837      0.182891      0.226240    0.00511605   -0.00189927    0.00806693       1.01157       Opttag_D
      17       76.7542       1.03443       1.00000  0.000000E+00       1.00000  0.000000E+00      0.928189       71.0006       1.00000       33.7565  0.000000E+00       8.93363  0.000000E+00       8.93363  0.000000E+00  0.000000E+00      -17.8673       78.9652       3.74714        1       Scatag_D
      17       1827.88       5577.26  0.000000E+00  0.000000E+00       2389.22  0.000000E+00       2640.00       6659.36  0.000000E+00       11403.9       55.7850       9097.52 -1.000000E+10 -1.000000E+10 -1.000000E+10 -1.000000E+10 -1.000000E+10  0.000000E+00  0.000000E+00  0.000000E+00  0.000000E+00  0.000000E+00  0.000000E+00  0.000000E+00  0.000000E+00  0.000000E+00  0.000000E+00  0.000000E+00  0.000000E+00       Funtag_D
      18  4.416211E+05  4.019062E+05       39714.9       10.3975     0.0213830      0.222405      0.182824      0.226253    0.00538197   -0.00205432    0.00808401       1.01269       Opttag_D
      18       76.7622       1.03309       1.00000  0.000000E+00       1.00000  0.000000E+00      0.928107       71.0572       1.00000       33.7191  0.000000E+00       8.93270  0.000000E+00       8.93270  0.000000E+00  0.000000E+00      -17.8654       78.9652       3.74714        1       Scatag_D
      18       1835.63       5589.62  0.000000E+00  0.000000E+00       2390.91  0.000000E+00       2642.61       6672.28  0.000000E+00       11427.4       56.0406       9100.40 -1.000000E+10 -1.000000E+10 -1.000000E+10 -1.000000E+10 -1.000000E+10  0.000000E+00  0.000000E+00  0.000000E+00  0.000000E+00  0.000000E+00  0.000000E+00  0.000000E+00  0.000000E+00  0.000000E+00  0.000000E+00  0.000000E+00  0.000000E+00       Funtag_D
      19  4.415780E+05  4.017508E+05       39827.2       17.7300     0.0239194      0.260510      0.182718      0.226275    0.00579885   -0.00228645    0.00810867       1.01456       Opttag_D
      19       76.7751       1.03106       1.00000  0.000000E+00       1.00000  0.000000E+00      0.927971       71.1524       1.00000       33.6586  0.000000E+00       8.93062  0.000000E+00       8.93062  0.000000E+00  0.000000E+00      -17.8612       78.9652       3.74714        1       Scatag_D
      19       1846.85       5610.29  0.000000E+00  0.000000E+00       2393.91  0.000000E+00       2647.65       6699.97  0.000000E+00       11466.5       56.1759       9105.84 -1.000000E+10 -1.000000E+10 -1.000000E+10 -1.000000E+10 -1.000000E+10  0.000000E+00  0.000000E+00  0.000000E+00  0.000000E+00  0.000000E+00  0.000000E+00  0.000000E+00  0.000000E+00  0.000000E+00  0.000000E+00  0.000000E+00  0.000000E+00       Funtag_D
      20  4.415307E+05  4.016226E+05       39908.1       10.5843     0.0262230      0.295381      0.182629      0.226303    0.00614259   -0.00256574    0.00813270       1.01563       Opttag_D
      20       76.7867       1.02943       1.00000  0.000000E+00       1.00000  0.000000E+00      0.927851       71.2341       1.00000       33.6087  0.000000E+00       8.92850  0.000000E+00       8.92850  0.000000E+00  0.000000E+00      -17.8570       78.9652       3.74714        1       Scatag_D
      20       1857.81       5622.15  0.000000E+00  0.000000E+00       2396.82  0.000000E+00       2653.11       6704.15  0.000000E+00       11502.3       56.4297       9115.34 -1.000000E+10 -1.000000E+10 -1.000000E+10 -1.000000E+10 -1.000000E+10  0.000000E+00  0.000000E+00  0.000000E+00  0.000000E+00  0.000000E+00  0.000000E+00  0.000000E+00  0.000000E+00  0.000000E+00  0.000000E+00  0.000000E+00  0.000000E+00       Funtag_D
      21  4.414984E+05  4.015785E+05       39919.9       8.12653     0.0269751      0.309420      0.182598      0.226314    0.00626091   -0.00266133    0.00813400       1.01568       Opttag_D
      21       76.7899       1.02882       1.00000  0.000000E+00       1.00000  0.000000E+00      0.927815       71.2605       1.00000       33.5901  0.000000E+00       8.92748  0.000000E+00       8.92748  0.000000E+00  0.000000E+00      -17.8550       78.9652       3.74714        1       Scatag_D
      21       1858.40       5622.72  0.000000E+00  0.000000E+00       2397.99  0.000000E+00       2654.72       6701.83  0.000000E+00       11511.7       56.5681       9115.94 -1.000000E+10 -1.000000E+10 -1.000000E+10 -1.000000E+10 -1.000000E+10  0.000000E+00  0.000000E+00  0.000000E+00  0.000000E+00  0.000000E+00  0.000000E+00  0.000000E+00  0.000000E+00  0.000000E+00  0.000000E+00  0.000000E+00  0.000000E+00       Funtag_D
      22  4.414588E+05  4.015053E+05       39953.5       10.9554     0.0282616      0.335236      0.182551      0.226333    0.00645713   -0.00282840    0.00814181       1.01605       Opttag_D
      22       76.7949       1.02778       1.00000  0.000000E+00       1.00000  0.000000E+00      0.927755       71.3038       1.00000       33.5584  0.000000E+00       8.92554  0.000000E+00       8.92554  0.000000E+00  0.000000E+00      -17.8511       78.9652       3.74714        1       Scatag_D
      22       1861.97       5626.84  0.000000E+00  0.000000E+00       2400.01  0.000000E+00       2658.11       6704.15  0.000000E+00       11526.2       57.0042       9119.19 -1.000000E+10 -1.000000E+10 -1.000000E+10 -1.000000E+10 -1.000000E+10  0.000000E+00  0.000000E+00  0.000000E+00  0.000000E+00  0.000000E+00  0.000000E+00  0.000000E+00  0.000000E+00  0.000000E+00  0.000000E+00  0.000000E+00  0.000000E+00       Funtag_D
      23  4.414332E+05  4.014157E+05       40017.5       14.6730     0.0299442      0.367645      0.182493      0.226359    0.00669745   -0.00306206    0.00816051       1.01676       Opttag_D
      23       76.8017       1.02641       1.00000  0.000000E+00       1.00000  0.000000E+00      0.927681       71.3529       1.00000       33.5232  0.000000E+00       8.92365  0.000000E+00       8.92365  0.000000E+00  0.000000E+00      -17.8473       78.9652       3.74714        1       Scatag_D
      23       1870.54       5634.66  0.000000E+00  0.000000E+00       2402.47  0.000000E+00       2663.03       6715.84  0.000000E+00       11545.6       57.4880       9127.82 -1.000000E+10 -1.000000E+10 -1.000000E+10 -1.000000E+10 -1.000000E+10  0.000000E+00  0.000000E+00  0.000000E+00  0.000000E+00  0.000000E+00  0.000000E+00  0.000000E+00  0.000000E+00  0.000000E+00  0.000000E+00  0.000000E+00  0.000000E+00       Funtag_D
      24  4.414042E+05  4.013978E+05       40006.5       8.78088     0.0302369      0.374977      0.182482      0.226361    0.00674561   -0.00310751    0.00815976       1.01656       Opttag_D
      24       76.8024       1.02602       1.00000  0.000000E+00       1.00000  0.000000E+00      0.927672       71.3574       1.00000       33.5180  0.000000E+00       8.92328  0.000000E+00       8.92328  0.000000E+00  0.000000E+00      -17.8466       78.9652       3.74714        1       Scatag_D
      24       1870.19       5632.50  0.000000E+00  0.000000E+00       2402.93  0.000000E+00       2663.44       6705.41  0.000000E+00       11547.2       57.5098       9127.32 -1.000000E+10 -1.000000E+10 -1.000000E+10 -1.000000E+10 -1.000000E+10  0.000000E+00  0.000000E+00  0.000000E+00  0.000000E+00  0.000000E+00  0.000000E+00  0.000000E+00  0.000000E+00  0.000000E+00  0.000000E+00  0.000000E+00  0.000000E+00       Funtag_D
      25  4.413702E+05  4.013433E+05       40026.9       8.16072     0.0312920      0.397329      0.182450      0.226376    0.00689157   -0.00326400    0.00816714       1.01679       Opttag_D
      25       76.8062       1.02497       1.00000  0.000000E+00       1.00000  0.000000E+00      0.927631       71.3793       1.00000       33.5016  0.000000E+00       8.92228  0.000000E+00       8.92228  0.000000E+00  0.000000E+00      -17.8446       78.9652       3.74714        1       Scatag_D
      25       1873.58       5634.95  0.000000E+00  0.000000E+00       2404.43  0.000000E+00       2665.19       6705.25  0.000000E+00       11556.9       57.4969       9129.11 -1.000000E+10 -1.000000E+10 -1.000000E+10 -1.000000E+10 -1.000000E+10  0.000000E+00  0.000000E+00  0.000000E+00  0.000000E+00  0.000000E+00  0.000000E+00  0.000000E+00  0.000000E+00  0.000000E+00  0.000000E+00  0.000000E+00  0.000000E+00       Funtag_D
      26  4.413449E+05  4.012943E+05       40050.6       8.88140     0.0324224      0.421137      0.182420      0.226394    0.00702324   -0.00344056    0.00817370       1.01700       Opttag_D
      26       76.8103       1.02407       1.00000  0.000000E+00       1.00000  0.000000E+00      0.927586       71.4045       1.00000       33.4863  0.000000E+00       8.92109  0.000000E+00       8.92109  0.000000E+00  0.000000E+00      -17.8422       78.9652       3.74714        1       Scatag_D
      26       1876.59       5637.36  0.000000E+00  0.000000E+00       2406.07  0.000000E+00       2666.87       6706.54  0.000000E+00       11567.5       57.3330       9132.38 -1.000000E+10 -1.000000E+10 -1.000000E+10 -1.000000E+10 -1.000000E+10  0.000000E+00  0.000000E+00  0.000000E+00  0.000000E+00  0.000000E+00  0.000000E+00  0.000000E+00  0.000000E+00  0.000000E+00  0.000000E+00  0.000000E+00  0.000000E+00       Funtag_D
      27  4.413184E+05  4.011918E+05       40126.6       18.7592     0.0356236      0.488880      0.182360      0.226448    0.00729806   -0.00391447    0.00818469       1.01778       Opttag_D
      27       76.8217       1.02244       1.00000  0.000000E+00       1.00000  0.000000E+00      0.927457       71.4828       1.00000       33.4506  0.000000E+00       8.91757  0.000000E+00       8.91757  0.000000E+00  0.000000E+00      -17.8351       78.9652       3.74714        1       Scatag_D
      27       1881.64       5645.98  0.000000E+00  0.000000E+00       2410.90  0.000000E+00       2670.89       6718.60  0.000000E+00       11596.0       56.9327       9145.61 -1.000000E+10 -1.000000E+10 -1.000000E+10 -1.000000E+10 -1.000000E+10  0.000000E+00  0.000000E+00  0.000000E+00  0.000000E+00  0.000000E+00  0.000000E+00  0.000000E+00  0.000000E+00  0.000000E+00  0.000000E+00  0.000000E+00  0.000000E+00       Funtag_D
      28  4.412772E+05  4.011758E+05       40101.5       8.09288     0.0363351      0.504482      0.182351      0.226465    0.00734099   -0.00404684    0.00817704       1.01736       Opttag_D
      28       76.8241       1.02222       1.00000  0.000000E+00       1.00000  0.000000E+00      0.927430       71.5006       1.00000       33.4443  0.000000E+00       8.91682  0.000000E+00       8.91682  0.000000E+00  0.000000E+00      -17.8336       78.9652       3.74714        1       Scatag_D
      28       1878.12       5641.32  0.000000E+00  0.000000E+00       2412.01  0.000000E+00       2670.59       6695.11  0.000000E+00       11601.2       56.9264       9146.20 -1.000000E+10 -1.000000E+10 -1.000000E+10 -1.000000E+10 -1.000000E+10  0.000000E+00  0.000000E+00  0.000000E+00  0.000000E+00  0.000000E+00  0.000000E+00  0.000000E+00  0.000000E+00  0.000000E+00  0.000000E+00  0.000000E+00  0.000000E+00       Funtag_D
      29  4.412592E+05  4.011793E+05       40079.9       7.01530     0.0365228      0.510152      0.182353      0.226471    0.00733155   -0.00407982    0.00816588       1.01700       Opttag_D
      29       76.8245       1.02237       1.00000  0.000000E+00       1.00000  0.000000E+00      0.927423       71.5085       1.00000       33.4430  0.000000E+00       8.91667  0.000000E+00       8.91667  0.000000E+00  0.000000E+00      -17.8333       78.9652       3.74714        1       Scatag_D
      29       1873.00       5637.37  0.000000E+00  0.000000E+00       2412.41  0.000000E+00       2668.69       6688.77  0.000000E+00       11600.4       57.0211       9142.27 -1.000000E+10 -1.000000E+10 -1.000000E+10 -1.000000E+10 -1.000000E+10  0.000000E+00  0.000000E+00  0.000000E+00  0.000000E+00  0.000000E+00  0.000000E+00  0.000000E+00  0.000000E+00  0.000000E+00  0.000000E+00  0.000000E+00  0.000000E+00       Funtag_D
      30  4.412321E+05  4.011682E+05       40063.9       8.08934     0.0375571      0.535319      0.182346      0.226494    0.00736128   -0.00423117    0.00815502       1.01668       Opttag_D
      30       76.8276       1.02240       1.00000  0.000000E+00       1.00000  0.000000E+00      0.927383       71.5404       1.00000       33.4333  0.000000E+00       8.91595  0.000000E+00       8.91595  0.000000E+00  0.000000E+00      -17.8319       78.9652       3.74714        1       Scatag_D
      30       1868.02       5633.84  0.000000E+00  0.000000E+00       2414.27  0.000000E+00       2666.08       6680.55  0.000000E+00       11603.9       57.2699       9139.92 -1.000000E+10 -1.000000E+10 -1.000000E+10 -1.000000E+10 -1.000000E+10  0.000000E+00  0.000000E+00  0.000000E+00  0.000000E+00  0.000000E+00  0.000000E+00  0.000000E+00  0.000000E+00  0.000000E+00  0.000000E+00  0.000000E+00  0.000000E+00       Funtag_D
      31  4.412212E+05  4.011222E+05       40099.0       20.0615     0.0399149      0.590243      0.182320      0.226536    0.00748476   -0.00458166    0.00815222       1.01682       Opttag_D
      31       76.8348       1.02189       1.00000  0.000000E+00       1.00000  0.000000E+00      0.927304       71.6026       1.00000       33.4112  0.000000E+00       8.91519  0.000000E+00       8.91519  0.000000E+00  0.000000E+00      -17.8304       78.9652       3.74714        1       Scatag_D
      31       1866.74       5635.39  0.000000E+00  0.000000E+00       2418.34  0.000000E+00       2663.73       6701.20  0.000000E+00       11613.8       57.4349       9142.36 -1.000000E+10 -1.000000E+10 -1.000000E+10 -1.000000E+10 -1.000000E+10  0.000000E+00  0.000000E+00  0.000000E+00  0.000000E+00  0.000000E+00  0.000000E+00  0.000000E+00  0.000000E+00  0.000000E+00  0.000000E+00  0.000000E+00  0.000000E+00       Funtag_D
      32  4.411856E+05  4.011144E+05       40071.2       8.12352     0.0403331      0.599673      0.182315      0.226544    0.00750568   -0.00463714    0.00815110       1.01666       Opttag_D
      32       76.8361       1.02181       1.00000  0.000000E+00       1.00000  0.000000E+00      0.927289       71.6144       1.00000       33.4071  0.000000E+00       8.91490  0.000000E+00       8.91490  0.000000E+00  0.000000E+00      -17.8298       78.9652       3.74714        1       Scatag_D
      32       1866.23       5633.61  0.000000E+00  0.000000E+00       2419.11  0.000000E+00       2663.76       6672.04  0.000000E+00       11615.8       57.4264       9143.20 -1.000000E+10 -1.000000E+10 -1.000000E+10 -1.000000E+10 -1.000000E+10  0.000000E+00  0.000000E+00  0.000000E+00  0.000000E+00  0.000000E+00  0.000000E+00  0.000000E+00  0.000000E+00  0.000000E+00  0.000000E+00  0.000000E+00  0.000000E+00       Funtag_D
      33  4.411696E+05  4.010943E+05       40075.3       5.95157     0.0410211      0.615128      0.182304      0.226555    0.00755961   -0.00473454    0.00815294       1.01673       Opttag_D
      33       76.8383       1.02148       1.00000  0.000000E+00       1.00000  0.000000E+00      0.927267       71.6313       1.00000       33.4001  0.000000E+00       8.91471  0.000000E+00       8.91471  0.000000E+00  0.000000E+00      -17.8294       78.9652       3.74714        1       Scatag_D
      33       1867.07       5634.33  0.000000E+00  0.000000E+00       2420.30  0.000000E+00       2663.98       6669.52  0.000000E+00       11618.9       57.2952       9143.89 -1.000000E+10 -1.000000E+10 -1.000000E+10 -1.000000E+10 -1.000000E+10  0.000000E+00  0.000000E+00  0.000000E+00  0.000000E+00  0.000000E+00  0.000000E+00  0.000000E+00  0.000000E+00  0.000000E+00  0.000000E+00  0.000000E+00  0.000000E+00       Funtag_D
      34  4.411513E+05  4.010580E+05       40093.3       7.24849     0.0423408      0.644919      0.182284      0.226575    0.00765686   -0.00493404    0.00815903       1.01696       Opttag_D
      34       76.8426       1.02091       1.00000  0.000000E+00       1.00000  0.000000E+00      0.927227       71.6641       1.00000       33.3877  0.000000E+00       8.91436  0.000000E+00       8.91436  0.000000E+00  0.000000E+00      -17.8287       78.9652       3.74714        1       Scatag_D
      34       1869.86       5636.85  0.000000E+00  0.000000E+00       2422.57  0.000000E+00       2664.36       6672.11  0.000000E+00       11624.7       57.0458       9145.84 -1.000000E+10 -1.000000E+10 -1.000000E+10 -1.000000E+10 -1.000000E+10  0.000000E+00  0.000000E+00  0.000000E+00  0.000000E+00  0.000000E+00  0.000000E+00  0.000000E+00  0.000000E+00  0.000000E+00  0.000000E+00  0.000000E+00  0.000000E+00       Funtag_D
      35  4.411226E+05  4.010025E+05       40120.1       8.52648     0.0443521      0.690347      0.182254      0.226609    0.00780571   -0.00526411    0.00816573       1.01724       Opttag_D
      35       76.8494       1.02007       1.00000  0.000000E+00       1.00000  0.000000E+00      0.927167       71.7173       1.00000       33.3705  0.000000E+00       8.91358  0.000000E+00       8.91358  0.000000E+00  0.000000E+00      -17.8272       78.9652       3.74714        1       Scatag_D
      35       1872.93       5640.02  0.000000E+00  0.000000E+00       2426.06  0.000000E+00       2665.02       6678.15  0.000000E+00       11632.8       56.7266       9148.40 -1.000000E+10 -1.000000E+10 -1.000000E+10 -1.000000E+10 -1.000000E+10  0.000000E+00  0.000000E+00  0.000000E+00  0.000000E+00  0.000000E+00  0.000000E+00  0.000000E+00  0.000000E+00  0.000000E+00  0.000000E+00  0.000000E+00  0.000000E+00       Funtag_D
      36  4.411045E+05  4.009492E+05       40155.3       11.2141     0.0464095      0.736118      0.182226      0.226648    0.00794878   -0.00562147    0.00817333       1.01759       Opttag_D
      36       76.8565       1.01930       1.00000  0.000000E+00       1.00000  0.000000E+00      0.927108       71.7715       1.00000       33.3559  0.000000E+00       8.91291  0.000000E+00       8.91291  0.000000E+00  0.000000E+00      -17.8258       78.9652       3.74714        2       Scatag_D
      36       1876.42       5643.92  0.000000E+00  0.000000E+00       2429.61  0.000000E+00       2665.50       6690.44  0.000000E+00       11641.1       56.6502       9151.69 -1.000000E+10 -1.000000E+10 -1.000000E+10 -1.000000E+10 -1.000000E+10  0.000000E+00  0.000000E+00  0.000000E+00  0.000000E+00  0.000000E+00  0.000000E+00  0.000000E+00  0.000000E+00  0.000000E+00  0.000000E+00  0.000000E+00  0.000000E+00       Funtag_D
      37  4.410798E+05  4.009068E+05       40172.9       6.56544     0.0479973      0.771312      0.182205      0.226678    0.00806234   -0.00591241    0.00817827       1.01787       Opttag_D
      37       76.8622       1.01872       1.00000  0.000000E+00       1.00000  0.000000E+00      0.927064       71.8156       1.00000       33.3458  0.000000E+00       8.91235  0.000000E+00       8.91235  0.000000E+00  0.000000E+00      -17.8247       78.9652       3.74714        1       Scatag_D
      37       1878.69       5646.97  0.000000E+00  0.000000E+00       2432.38  0.000000E+00       2665.94       6691.64  0.000000E+00       11646.8       56.6441       9153.90 -1.000000E+10 -1.000000E+10 -1.000000E+10 -1.000000E+10 -1.000000E+10  0.000000E+00  0.000000E+00  0.000000E+00  0.000000E+00  0.000000E+00  0.000000E+00  0.000000E+00  0.000000E+00  0.000000E+00  0.000000E+00  0.000000E+00  0.000000E+00       Funtag_D
      38  4.410592E+05  4.008724E+05       40186.8       6.19704     0.0491490      0.796813      0.182188      0.226703    0.00815460   -0.00613903    0.00817936       1.01806       Opttag_D
      38       76.8665       1.01825       1.00000  0.000000E+00       1.00000  0.000000E+00      0.927036       71.8497       1.00000       33.3396  0.000000E+00       8.91206  0.000000E+00       8.91206  0.000000E+00  0.000000E+00      -17.8241       78.9652       3.74714        1       Scatag_D
      38       1879.19       5649.14  0.000000E+00  0.000000E+00       2434.46  0.000000E+00       2665.63       6699.00  0.000000E+00       11649.5       56.7797       9153.07 -1.000000E+10 -1.000000E+10 -1.000000E+10 -1.000000E+10 -1.000000E+10  0.000000E+00  0.000000E+00  0.000000E+00  0.000000E+00  0.000000E+00  0.000000E+00  0.000000E+00  0.000000E+00  0.000000E+00  0.000000E+00  0.000000E+00  0.000000E+00       Funtag_D
      39  4.410358E+05  4.008246E+05       40211.2       8.69716     0.0507564      0.831570      0.182164      0.226736    0.00828292   -0.00644551    0.00818178       1.01844       Opttag_D
      39       76.8725       1.01764       1.00000  0.000000E+00       1.00000  0.000000E+00      0.926997       71.9001       1.00000       33.3330  0.000000E+00       8.91194  0.000000E+00       8.91194  0.000000E+00  0.000000E+00      -17.8239       78.9652       3.74714        1       Scatag_D
      39       1880.30       5653.36  0.000000E+00  0.000000E+00       2437.37  0.000000E+00       2665.19       6713.51  0.000000E+00       11652.9       56.9368       9151.59 -1.000000E+10 -1.000000E+10 -1.000000E+10 -1.000000E+10 -1.000000E+10  0.000000E+00  0.000000E+00  0.000000E+00  0.000000E+00  0.000000E+00  0.000000E+00  0.000000E+00  0.000000E+00  0.000000E+00  0.000000E+00  0.000000E+00  0.000000E+00       Funtag_D
      40  4.410147E+05  4.007804E+05       40234.4       10.1536     0.0522706      0.863059      0.182143      0.226764    0.00840148   -0.00670438    0.00818576       1.01894       Opttag_D
      40       76.8779       1.01707       1.00000  0.000000E+00       1.00000  0.000000E+00      0.926964       71.9472       1.00000       33.3297  0.000000E+00       8.91218  0.000000E+00       8.91218  0.000000E+00  0.000000E+00      -17.8244       78.9652       3.74714        1       Scatag_D
      40       1882.13       5658.89  0.000000E+00  0.000000E+00       2440.16  0.000000E+00       2665.11       6724.62  0.000000E+00       11655.7       56.9109       9150.84 -1.000000E+10 -1.000000E+10 -1.000000E+10 -1.000000E+10 -1.000000E+10  0.000000E+00  0.000000E+00  0.000000E+00  0.000000E+00  0.000000E+00  0.000000E+00  0.000000E+00  0.000000E+00  0.000000E+00  0.000000E+00  0.000000E+00  0.000000E+00       Funtag_D
      41  4.409949E+05  4.007692E+05       40225.7       6.90961     0.0525278      0.867587      0.182138      0.226767    0.00843145   -0.00673935    0.00818418       1.01896       Opttag_D
      41       76.8786       1.01690       1.00000  0.000000E+00       1.00000  0.000000E+00      0.926961       71.9576       1.00000       33.3304  0.000000E+00       8.91264  0.000000E+00       8.91264  0.000000E+00  0.000000E+00      -17.8253       78.9652       3.74714        1       Scatag_D
      41       1881.41       5659.05  0.000000E+00  0.000000E+00       2440.67  0.000000E+00       2664.88       6719.40  0.000000E+00       11655.0       56.7622       9148.54 -1.000000E+10 -1.000000E+10 -1.000000E+10 -1.000000E+10 -1.000000E+10  0.000000E+00  0.000000E+00  0.000000E+00  0.000000E+00  0.000000E+00  0.000000E+00  0.000000E+00  0.000000E+00  0.000000E+00  0.000000E+00  0.000000E+00  0.000000E+00       Funtag_D
      42  4.409704E+05  4.007502E+05       40220.3       6.68079     0.0530605      0.876384      0.182129      0.226771    0.00848250   -0.00678859    0.00818369       1.01904       Opttag_D
      42       76.8799       1.01662       1.00000  0.000000E+00       1.00000  0.000000E+00      0.926951       71.9783       1.00000       33.3321  0.000000E+00       8.91358  0.000000E+00       8.91358  0.000000E+00  0.000000E+00      -17.8272       78.9652       3.74714        1       Scatag_D
      42       1881.18       5659.94  0.000000E+00  0.000000E+00       2441.66  0.000000E+00       2664.94       6716.74  0.000000E+00       11654.1       56.4609       9145.21 -1.000000E+10 -1.000000E+10 -1.000000E+10 -1.000000E+10 -1.000000E+10  0.000000E+00  0.000000E+00  0.000000E+00  0.000000E+00  0.000000E+00  0.000000E+00  0.000000E+00  0.000000E+00  0.000000E+00  0.000000E+00  0.000000E+00  0.000000E+00       Funtag_D
      43  4.409513E+05  4.007259E+05       40225.4       8.28821     0.0539042      0.890819      0.182119      0.226778    0.00854759   -0.00688970    0.00818361       1.01922       Opttag_D
      43       76.8819       1.01627       1.00000  0.000000E+00       1.00000  0.000000E+00      0.926931       72.0084       1.00000       33.3334  0.000000E+00       8.91444  0.000000E+00       8.91444  0.000000E+00  0.000000E+00      -17.8289       78.9652       3.74714        1       Scatag_D
      43       1881.14       5662.02  0.000000E+00  0.000000E+00       2443.19  0.000000E+00       2665.22       6719.55  0.000000E+00       11654.3       56.3908       9143.63 -1.000000E+10 -1.000000E+10 -1.000000E+10 -1.000000E+10 -1.000000E+10  0.000000E+00  0.000000E+00  0.000000E+00  0.000000E+00  0.000000E+00  0.000000E+00  0.000000E+00  0.000000E+00  0.000000E+00  0.000000E+00  0.000000E+00  0.000000E+00       Funtag_D
      44  4.409244E+05  4.006855E+05       40238.9       7.37200     0.0555314      0.918507      0.182101      0.226801    0.00865593   -0.00712851    0.00818386       1.01965       Opttag_D
      44       76.8858       1.01570       1.00000  0.000000E+00       1.00000  0.000000E+00      0.926893       72.0628       1.00000       33.3356  0.000000E+00       8.91578  0.000000E+00       8.91578  0.000000E+00  0.000000E+00      -17.8316       78.9652       3.74714        1       Scatag_D
      44       1881.26       5666.78  0.000000E+00  0.000000E+00       2446.01  0.000000E+00       2665.89       6722.94  0.000000E+00       11656.0       56.5207       9143.46 -1.000000E+10 -1.000000E+10 -1.000000E+10 -1.000000E+10 -1.000000E+10  0.000000E+00  0.000000E+00  0.000000E+00  0.000000E+00  0.000000E+00  0.000000E+00  0.000000E+00  0.000000E+00  0.000000E+00  0.000000E+00  0.000000E+00  0.000000E+00       Funtag_D
      45  4.409020E+05  4.006449E+05       40257.1       9.13651     0.0572095      0.946107      0.182084      0.226829    0.00876481   -0.00740464    0.00818326       1.02010       Opttag_D
      45       76.8894       1.01507       1.00000  0.000000E+00       1.00000  0.000000E+00      0.926850       72.1163       1.00000       33.3384  0.000000E+00       8.91725  0.000000E+00       8.91725  0.000000E+00  0.000000E+00      -17.8345       78.9652       3.74714        1       Scatag_D
      45       1880.98       5671.71  0.000000E+00  0.000000E+00       2448.85  0.000000E+00       2665.49       6733.24  0.000000E+00       11657.9       56.7936       9142.15 -1.000000E+10 -1.000000E+10 -1.000000E+10 -1.000000E+10 -1.000000E+10  0.000000E+00  0.000000E+00  0.000000E+00  0.000000E+00  0.000000E+00  0.000000E+00  0.000000E+00  0.000000E+00  0.000000E+00  0.000000E+00  0.000000E+00  0.000000E+00       Funtag_D
      46  4.408804E+05  4.006146E+05       40265.8       6.57304     0.0584760      0.966199      0.182070      0.226856    0.00884606   -0.00762836    0.00818297       1.02032       Opttag_D
      46       76.8915       1.01454       1.00000  0.000000E+00       1.00000  0.000000E+00      0.926819       72.1538       1.00000       33.3408  0.000000E+00       8.91835  0.000000E+00       8.91835  0.000000E+00  0.000000E+00      -17.8367       78.9652       3.74714        1       Scatag_D
      46       1880.85       5674.20  0.000000E+00  0.000000E+00       2451.02  0.000000E+00       2664.47       6738.93  0.000000E+00       11659.1       57.0143       9140.22 -1.000000E+10 -1.000000E+10 -1.000000E+10 -1.000000E+10 -1.000000E+10  0.000000E+00  0.000000E+00  0.000000E+00  0.000000E+00  0.000000E+00  0.000000E+00  0.000000E+00  0.000000E+00  0.000000E+00  0.000000E+00  0.000000E+00  0.000000E+00       Funtag_D
      47  4.408631E+05  4.005941E+05       40269.0       6.29210     0.0592959      0.978091      0.182058      0.226881    0.00890101   -0.00781503    0.00818152       1.02046       Opttag_D
      47       76.8923       1.01412       1.00000  0.000000E+00       1.00000  0.000000E+00      0.926799       72.1762       1.00000       33.3428  0.000000E+00       8.91928  0.000000E+00       8.91928  0.000000E+00  0.000000E+00      -17.8386       78.9652       3.74714        1       Scatag_D
      47       1880.18       5675.76  0.000000E+00  0.000000E+00       2452.50  0.000000E+00       2663.08       6744.15  0.000000E+00       11659.1       57.0212       9137.19 -1.000000E+10 -1.000000E+10 -1.000000E+10 -1.000000E+10 -1.000000E+10  0.000000E+00  0.000000E+00  0.000000E+00  0.000000E+00  0.000000E+00  0.000000E+00  0.000000E+00  0.000000E+00  0.000000E+00  0.000000E+00  0.000000E+00  0.000000E+00       Funtag_D
      48  4.408414E+05  4.005630E+05       40278.4       8.07161     0.0607228      0.997532      0.182045      0.226928    0.00898437   -0.00813977    0.00817907       1.02080       Opttag_D
      48       76.8933       1.01348       1.00000  0.000000E+00       1.00000  0.000000E+00      0.926764       72.2151       1.00000       33.3486  0.000000E+00       8.92090  0.000000E+00       8.92090  0.000000E+00  0.000000E+00      -17.8418       78.9652       3.74714        1       Scatag_D
      48       1879.06       5679.52  0.000000E+00  0.000000E+00       2455.09  0.000000E+00       2661.43       6753.11  0.000000E+00       11659.4       56.7642       9134.07 -1.000000E+10 -1.000000E+10 -1.000000E+10 -1.000000E+10 -1.000000E+10  0.000000E+00  0.000000E+00  0.000000E+00  0.000000E+00  0.000000E+00  0.000000E+00  0.000000E+00  0.000000E+00  0.000000E+00  0.000000E+00  0.000000E+00  0.000000E+00       Funtag_D
      49  4.408198E+05  4.005377E+05       40282.1       8.36661     0.0623549       1.01935      0.182037      0.226987    0.00905235   -0.00849956    0.00817518       1.02114       Opttag_D
      49       76.8948       1.01303       1.00000  0.000000E+00       1.00000  0.000000E+00      0.926727       72.2615       1.00000       33.3573  0.000000E+00       8.92249  0.000000E+00       8.92249  0.000000E+00  0.000000E+00      -17.8450       78.9652       3.74714        1       Scatag_D
      49       1877.27       5683.28  0.000000E+00  0.000000E+00       2458.08  0.000000E+00       2660.95       6752.77  0.000000E+00       11660.5       56.4325       9132.84 -1.000000E+10 -1.000000E+10 -1.000000E+10 -1.000000E+10 -1.000000E+10  0.000000E+00  0.000000E+00  0.000000E+00  0.000000E+00  0.000000E+00  0.000000E+00  0.000000E+00  0.000000E+00  0.000000E+00  0.000000E+00  0.000000E+00  0.000000E+00       Funtag_D
      50  4.407989E+05  4.005235E+05       40275.4       6.77148     0.0634015       1.03235      0.182033      0.227033    0.00909044   -0.00877343    0.00817115       1.02116       Opttag_D
      50       76.8957       1.01277       1.00000  0.000000E+00       1.00000  0.000000E+00      0.926706       72.2899       1.00000       33.3643  0.000000E+00       8.92350  0.000000E+00       8.92350  0.000000E+00  0.000000E+00      -17.8470       78.9652       3.74714        1       Scatag_D
      50       1875.42       5683.55  0.000000E+00  0.000000E+00       2460.04  0.000000E+00       2660.81       6747.29  0.000000E+00       11660.7       56.2635       9131.32 -1.000000E+10 -1.000000E+10 -1.000000E+10 -1.000000E+10 -1.000000E+10  0.000000E+00  0.000000E+00  0.000000E+00  0.000000E+00  0.000000E+00  0.000000E+00  0.000000E+00  0.000000E+00  0.000000E+00  0.000000E+00  0.000000E+00  0.000000E+00       Funtag_D
      51  4.407752E+05  4.004947E+05       40280.6       7.94629     0.0653147       1.05628      0.182023      0.227113    0.00916771   -0.00928141    0.00817258       1.02132       Opttag_D
      51       76.8976       1.01223       1.00000  0.000000E+00       1.00000  0.000000E+00      0.926666       72.3423       1.00000       33.3769  0.000000E+00       8.92515  0.000000E+00       8.92515  0.000000E+00  0.000000E+00      -17.8503       78.9652       3.74714        1       Scatag_D
      51       1876.08       5685.32  0.000000E+00  0.000000E+00       2463.44  0.000000E+00       2660.82       6746.64  0.000000E+00       11661.5       56.3949       9130.38 -1.000000E+10 -1.000000E+10 -1.000000E+10 -1.000000E+10 -1.000000E+10  0.000000E+00  0.000000E+00  0.000000E+00  0.000000E+00  0.000000E+00  0.000000E+00  0.000000E+00  0.000000E+00  0.000000E+00  0.000000E+00  0.000000E+00  0.000000E+00       Funtag_D
      52  4.407583E+05  4.004619E+05       40296.4       9.64236     0.0674376       1.08257      0.182010      0.227204    0.00925575   -0.00982833    0.00818007       1.02156       Opttag_D
      52       76.8996       1.01156       1.00000  0.000000E+00       1.00000  0.000000E+00      0.926624       72.3961       1.00000       33.3907  0.000000E+00       8.92704  0.000000E+00       8.92704  0.000000E+00  0.000000E+00      -17.8541       78.9652       3.74714        1       Scatag_D
      52       1879.52       5688.03  0.000000E+00  0.000000E+00       2467.10  0.000000E+00       2660.84       6750.82  0.000000E+00       11662.7       56.7136       9130.75 -1.000000E+10 -1.000000E+10 -1.000000E+10 -1.000000E+10 -1.000000E+10  0.000000E+00  0.000000E+00  0.000000E+00  0.000000E+00  0.000000E+00  0.000000E+00  0.000000E+00  0.000000E+00  0.000000E+00  0.000000E+00  0.000000E+00  0.000000E+00       Funtag_D
      53  4.407392E+05  4.004436E+05       40295.6       6.56831     0.0681511       1.09107      0.182001      0.227239    0.00930461    -0.0100537    0.00818263       1.02150       Opttag_D
      53       76.9002       1.01114       1.00000  0.000000E+00       1.00000  0.000000E+00      0.926612       72.4120       1.00000       33.3943  0.000000E+00       8.92770  0.000000E+00       8.92770  0.000000E+00  0.000000E+00      -17.8554       78.9652       3.74714        1       Scatag_D
      53       1880.69       5687.33  0.000000E+00  0.000000E+00       2468.28  0.000000E+00       2660.16       6750.37  0.000000E+00       11662.4       56.9759       9129.42 -1.000000E+10 -1.000000E+10 -1.000000E+10 -1.000000E+10 -1.000000E+10  0.000000E+00  0.000000E+00  0.000000E+00  0.000000E+00  0.000000E+00  0.000000E+00  0.000000E+00  0.000000E+00  0.000000E+00  0.000000E+00  0.000000E+00  0.000000E+00       Funtag_D
      54  4.407125E+05  4.004007E+05       40311.8       6.29327     0.0700940       1.11439      0.181979      0.227330    0.00941974    -0.0106150    0.00819391       1.02158       Opttag_D
      54       76.9019       1.01015       1.00000  0.000000E+00       1.00000  0.000000E+00      0.926579       72.4534       1.00000       33.4055  0.000000E+00       8.92938  0.000000E+00       8.92938  0.000000E+00  0.000000E+00      -17.8588       78.9652       3.74714        1       Scatag_D
      54       1885.88       5688.24  0.000000E+00  0.000000E+00       2471.31  0.000000E+00       2658.37       6760.54  0.000000E+00       11663.1       57.4043       9126.96 -1.000000E+10 -1.000000E+10 -1.000000E+10 -1.000000E+10 -1.000000E+10  0.000000E+00  0.000000E+00  0.000000E+00  0.000000E+00  0.000000E+00  0.000000E+00  0.000000E+00  0.000000E+00  0.000000E+00  0.000000E+00  0.000000E+00  0.000000E+00       Funtag_D
      55  4.406951E+05  4.003718E+05       40323.3       9.71223     0.0723036       1.14071      0.181968      0.227432    0.00949732    -0.0111918    0.00819956       1.02167       Opttag_D
      55       76.9041       1.00951       1.00000  0.000000E+00       1.00000  0.000000E+00      0.926544       72.5007       1.00000       33.4217  0.000000E+00       8.93095  0.000000E+00       8.93095  0.000000E+00  0.000000E+00      -17.8619       78.9652       3.74714        1       Scatag_D
      55       1888.49       5689.19  0.000000E+00  0.000000E+00       2474.60  0.000000E+00       2656.72       6767.30  0.000000E+00       11665.4       57.4523       9124.12 -1.000000E+10 -1.000000E+10 -1.000000E+10 -1.000000E+10 -1.000000E+10  0.000000E+00  0.000000E+00  0.000000E+00  0.000000E+00  0.000000E+00  0.000000E+00  0.000000E+00  0.000000E+00  0.000000E+00  0.000000E+00  0.000000E+00  0.000000E+00       Funtag_D
      56  4.406727E+05  4.003517E+05       40321.0       7.14790     0.0742092       1.16303      0.181961      0.227521    0.00955126    -0.0116941    0.00820231       1.02161       Opttag_D
      56       76.9061       1.00906       1.00000  0.000000E+00       1.00000  0.000000E+00      0.926519       72.5374       1.00000       33.4375  0.000000E+00       8.93208  0.000000E+00       8.93208  0.000000E+00  0.000000E+00      -17.8642       78.9652       3.74714        1       Scatag_D
      56       1889.75       5688.54  0.000000E+00  0.000000E+00       2477.22  0.000000E+00       2655.04       6764.79  0.000000E+00       11667.7       57.3860       9120.52 -1.000000E+10 -1.000000E+10 -1.000000E+10 -1.000000E+10 -1.000000E+10  0.000000E+00  0.000000E+00  0.000000E+00  0.000000E+00  0.000000E+00  0.000000E+00  0.000000E+00  0.000000E+00  0.000000E+00  0.000000E+00  0.000000E+00  0.000000E+00       Funtag_D
      57  4.406461E+05  4.003406E+05       40305.5       8.09532     0.0767307       1.19177      0.181963      0.227638    0.00958092    -0.0123238    0.00819566       1.02139       Opttag_D
      57       76.9092       1.00883       1.00000  0.000000E+00       1.00000  0.000000E+00      0.926493       72.5811       1.00000       33.4615  0.000000E+00       8.93290  0.000000E+00       8.93290  0.000000E+00  0.000000E+00      -17.8658       78.9652       3.74714        1       Scatag_D
      57       1886.69       5686.07  0.000000E+00  0.000000E+00       2480.41  0.000000E+00       2652.88       6759.09  0.000000E+00       11670.0       57.2658       9113.06 -1.000000E+10 -1.000000E+10 -1.000000E+10 -1.000000E+10 -1.000000E+10  0.000000E+00  0.000000E+00  0.000000E+00  0.000000E+00  0.000000E+00  0.000000E+00  0.000000E+00  0.000000E+00  0.000000E+00  0.000000E+00  0.000000E+00  0.000000E+00       Funtag_D
      58  4.406250E+05  4.003414E+05       40283.6       8.40142     0.0785307       1.21167      0.181968      0.227727    0.00957889    -0.0127772    0.00818717       1.02112       Opttag_D
      58       76.9120       1.00890       1.00000  0.000000E+00       1.00000  0.000000E+00      0.926480       72.6110       1.00000       33.4797  0.000000E+00       8.93323  0.000000E+00       8.93323  0.000000E+00  0.000000E+00      -17.8665       78.9652       3.74714        1       Scatag_D
      58       1882.78       5683.09  0.000000E+00  0.000000E+00       2482.55  0.000000E+00       2651.64       6749.55  0.000000E+00       11670.4       57.3045       9106.29 -1.000000E+10 -1.000000E+10 -1.000000E+10 -1.000000E+10 -1.000000E+10  0.000000E+00  0.000000E+00  0.000000E+00  0.000000E+00  0.000000E+00  0.000000E+00  0.000000E+00  0.000000E+00  0.000000E+00  0.000000E+00  0.000000E+00  0.000000E+00       Funtag_D
      59  4.406054E+05  4.003436E+05       40261.8       7.19799     0.0793123       1.21960      0.181970      0.227763    0.00957288    -0.0129747    0.00817984       1.02085       Opttag_D
      59       76.9135       1.00899       1.00000  0.000000E+00       1.00000  0.000000E+00      0.926480       72.6209       1.00000       33.4877  0.000000E+00       8.93311  0.000000E+00       8.93311  0.000000E+00  0.000000E+00      -17.8662       78.9652       3.74714        1       Scatag_D
      59       1879.41       5680.10  0.000000E+00  0.000000E+00       2483.41  0.000000E+00       2650.82       6740.84  0.000000E+00       11668.8       57.4249       9101.01 -1.000000E+10 -1.000000E+10 -1.000000E+10 -1.000000E+10 -1.000000E+10  0.000000E+00  0.000000E+00  0.000000E+00  0.000000E+00  0.000000E+00  0.000000E+00  0.000000E+00  0.000000E+00  0.000000E+00  0.000000E+00  0.000000E+00  0.000000E+00       Funtag_D
      60  4.405739E+05  4.003421E+05       40231.8       9.33599     0.0815769       1.24217      0.181976      0.227876    0.00957686    -0.0135451    0.00817617       1.02043       Opttag_D
      60       76.9179       1.00905       1.00000  0.000000E+00       1.00000  0.000000E+00      0.926477       72.6511       1.00000       33.5086  0.000000E+00       8.93323  0.000000E+00       8.93323  0.000000E+00  0.000000E+00      -17.8665       78.9652       3.74714        1       Scatag_D
      60       1877.73       5675.40  0.000000E+00  0.000000E+00       2485.63  0.000000E+00       2648.65       6731.50  0.000000E+00       11664.6       57.8148       9090.52 -1.000000E+10 -1.000000E+10 -1.000000E+10 -1.000000E+10 -1.000000E+10  0.000000E+00  0.000000E+00  0.000000E+00  0.000000E+00  0.000000E+00  0.000000E+00  0.000000E+00  0.000000E+00  0.000000E+00  0.000000E+00  0.000000E+00  0.000000E+00       Funtag_D
      61  4.405536E+05  4.003279E+05       40225.7       10.8098     0.0832159       1.25864      0.181970      0.227965    0.00961510    -0.0139721    0.00818363       1.02014       Opttag_D
      61       76.9208       1.00874       1.00000  0.000000E+00       1.00000  0.000000E+00      0.926469       72.6748       1.00000       33.5215  0.000000E+00       8.93404  0.000000E+00       8.93404  0.000000E+00  0.000000E+00      -17.8681       78.9652       3.74714        1       Scatag_D
      61       1881.16       5672.20  0.000000E+00  0.000000E+00       2487.04  0.000000E+00       2646.34       6733.72  0.000000E+00       11662.9       57.9041       9084.43 -1.000000E+10 -1.000000E+10 -1.000000E+10 -1.000000E+10 -1.000000E+10  0.000000E+00  0.000000E+00  0.000000E+00  0.000000E+00  0.000000E+00  0.000000E+00  0.000000E+00  0.000000E+00  0.000000E+00  0.000000E+00  0.000000E+00  0.000000E+00       Funtag_D
      62  4.405352E+05  4.003163E+05       40218.9       7.38638     0.0830737       1.25646      0.181965      0.227955    0.00964625    -0.0139421    0.00818693       1.02001       Opttag_D
      62       76.9206       1.00847       1.00000  0.000000E+00       1.00000  0.000000E+00      0.926468       72.6721       1.00000       33.5170  0.000000E+00       8.93429  0.000000E+00       8.93429  0.000000E+00  0.000000E+00      -17.8686       78.9652       3.74714        1       Scatag_D
      62       1882.67       5670.81  0.000000E+00  0.000000E+00       2486.79  0.000000E+00       2645.08       6731.94  0.000000E+00       11661.6       57.8812       9082.20 -1.000000E+10 -1.000000E+10 -1.000000E+10 -1.000000E+10 -1.000000E+10  0.000000E+00  0.000000E+00  0.000000E+00  0.000000E+00  0.000000E+00  0.000000E+00  0.000000E+00  0.000000E+00  0.000000E+00  0.000000E+00  0.000000E+00  0.000000E+00       Funtag_D
      63  4.405086E+05  4.002941E+05       40214.5       5.82092     0.0841667       1.26640      0.181954      0.228011    0.00970580    -0.0142042    0.00819770       1.01977       Opttag_D
      63       76.9227       1.00800       1.00000  0.000000E+00       1.00000  0.000000E+00      0.926455       72.6899       1.00000       33.5207  0.000000E+00       8.93520  0.000000E+00       8.93520  0.000000E+00  0.000000E+00      -17.8704       78.9652       3.74714        1       Scatag_D
      63       1887.63       5668.05  0.000000E+00  0.000000E+00       2487.44  0.000000E+00       2641.97       6734.31  0.000000E+00       11660.4       57.8549       9076.80 -1.000000E+10 -1.000000E+10 -1.000000E+10 -1.000000E+10 -1.000000E+10  0.000000E+00  0.000000E+00  0.000000E+00  0.000000E+00  0.000000E+00  0.000000E+00  0.000000E+00  0.000000E+00  0.000000E+00  0.000000E+00  0.000000E+00  0.000000E+00       Funtag_D
      64  4.404893E+05  4.002836E+05       40205.7       7.40659     0.0856410       1.28005      0.181950      0.228089    0.00973382    -0.0145500    0.00820054       1.01948       Opttag_D
      64       76.9259       1.00787       1.00000  0.000000E+00       1.00000  0.000000E+00      0.926436       72.7150       1.00000       33.5296  0.000000E+00       8.93584  0.000000E+00       8.93584  0.000000E+00  0.000000E+00      -17.8717       78.9652       3.74714        1       Scatag_D
      64       1888.93       5664.87  0.000000E+00  0.000000E+00       2488.36  0.000000E+00       2639.48       6733.86  0.000000E+00       11660.7       57.9466       9071.58 -1.000000E+10 -1.000000E+10 -1.000000E+10 -1.000000E+10 -1.000000E+10  0.000000E+00  0.000000E+00  0.000000E+00  0.000000E+00  0.000000E+00  0.000000E+00  0.000000E+00  0.000000E+00  0.000000E+00  0.000000E+00  0.000000E+00  0.000000E+00       Funtag_D
      65  4.404672E+05  4.002753E+05       40191.9       8.96459     0.0880302       1.30138      0.181948      0.228221    0.00975606    -0.0150860    0.00819805       1.01912       Opttag_D
      65       76.9314       1.00788       1.00000  0.000000E+00       1.00000  0.000000E+00      0.926405       72.7546       1.00000       33.5460  0.000000E+00       8.93659  0.000000E+00       8.93659  0.000000E+00  0.000000E+00      -17.8732       78.9652       3.74714        1       Scatag_D
      65       1887.79       5660.87  0.000000E+00  0.000000E+00       2489.91  0.000000E+00       2636.40       6731.24  0.000000E+00       11662.2       58.1613       9065.27 -1.000000E+10 -1.000000E+10 -1.000000E+10 -1.000000E+10 -1.000000E+10  0.000000E+00  0.000000E+00  0.000000E+00  0.000000E+00  0.000000E+00  0.000000E+00  0.000000E+00  0.000000E+00  0.000000E+00  0.000000E+00  0.000000E+00  0.000000E+00       Funtag_D
      66  4.404478E+05  4.002764E+05       40171.4       6.48348     0.0885480       1.30506      0.181950      0.228242    0.00975310    -0.0151776    0.00818836       1.01896       Opttag_D
      66       76.9334       1.00801       1.00000  0.000000E+00       1.00000  0.000000E+00      0.926393       72.7636       1.00000       33.5487  0.000000E+00       8.93665  0.000000E+00       8.93665  0.000000E+00  0.000000E+00      -17.8733       78.9652       3.74714        1       Scatag_D
      66       1883.33       5659.04  0.000000E+00  0.000000E+00       2490.20  0.000000E+00       2636.09       6721.67  0.000000E+00       11662.3       57.7616       9061.04 -1.000000E+10 -1.000000E+10 -1.000000E+10 -1.000000E+10 -1.000000E+10  0.000000E+00  0.000000E+00  0.000000E+00  0.000000E+00  0.000000E+00  0.000000E+00  0.000000E+00  0.000000E+00  0.000000E+00  0.000000E+00  0.000000E+00  0.000000E+00       Funtag_D
      67  4.404316E+05  4.002740E+05       40157.6       5.97311     0.0888533       1.30619      0.181947      0.228249    0.00975958    -0.0152259    0.00818038       1.01885       Opttag_D
      67       76.9350       1.00805       1.00000  0.000000E+00       1.00000  0.000000E+00      0.926381       72.7688       1.00000       33.5491  0.000000E+00       8.93661  0.000000E+00       8.93661  0.000000E+00  0.000000E+00      -17.8732       78.9652       3.74714        1       Scatag_D
      67       1879.66       5657.88  0.000000E+00  0.000000E+00       2490.22  0.000000E+00       2636.47       6717.30  0.000000E+00       11661.6       57.5648       9056.89 -1.000000E+10 -1.000000E+10 -1.000000E+10 -1.000000E+10 -1.000000E+10  0.000000E+00  0.000000E+00  0.000000E+00  0.000000E+00  0.000000E+00  0.000000E+00  0.000000E+00  0.000000E+00  0.000000E+00  0.000000E+00  0.000000E+00  0.000000E+00       Funtag_D
      68  4.404100E+05  4.002635E+05       40146.6       6.98874     0.0896820       1.31017      0.181939      0.228276    0.00978783    -0.0153599    0.00816905       1.01877       Opttag_D
      68       76.9386       1.00799       1.00000  0.000000E+00       1.00000  0.000000E+00      0.926359       72.7822       1.00000       33.5514  0.000000E+00       8.93694  0.000000E+00       8.93694  0.000000E+00  0.000000E+00      -17.8739       78.9652       3.74714        1       Scatag_D
      68       1874.46       5657.03  0.000000E+00  0.000000E+00       2490.32  0.000000E+00       2637.76       6717.16  0.000000E+00       11660.8       57.4638       9051.64 -1.000000E+10 -1.000000E+10 -1.000000E+10 -1.000000E+10 -1.000000E+10  0.000000E+00  0.000000E+00  0.000000E+00  0.000000E+00  0.000000E+00  0.000000E+00  0.000000E+00  0.000000E+00  0.000000E+00  0.000000E+00  0.000000E+00  0.000000E+00       Funtag_D
      69  4.403965E+05  4.002424E+05       40154.1       10.9749     0.0904207       1.31161      0.181928      0.228279    0.00984434    -0.0154413    0.00816673       1.01893       Opttag_D
      69       76.9430       1.00767       1.00000  0.000000E+00       1.00000  0.000000E+00      0.926331       72.7928       1.00000       33.5525  0.000000E+00       8.93727  0.000000E+00       8.93727  0.000000E+00  0.000000E+00      -17.8745       78.9652       3.74714        1       Scatag_D
      69       1873.39       5658.77  0.000000E+00  0.000000E+00       2490.11  0.000000E+00       2640.44       6726.77  0.000000E+00       11659.1       57.3921       9048.09 -1.000000E+10 -1.000000E+10 -1.000000E+10 -1.000000E+10 -1.000000E+10  0.000000E+00  0.000000E+00  0.000000E+00  0.000000E+00  0.000000E+00  0.000000E+00  0.000000E+00  0.000000E+00  0.000000E+00  0.000000E+00  0.000000E+00  0.000000E+00       Funtag_D
      70  4.403782E+05  4.002296E+05       40148.6       5.56498     0.0905269       1.31130      0.181921      0.228273    0.00987876    -0.0154355    0.00816960       1.01879       Opttag_D
      70       76.9438       1.00736       1.00000  0.000000E+00       1.00000  0.000000E+00      0.926327       72.7934       1.00000       33.5523  0.000000E+00       8.93753  0.000000E+00       8.93753  0.000000E+00  0.000000E+00      -17.8751       78.9652       3.74714        1       Scatag_D
      70       1874.71       5657.19  0.000000E+00  0.000000E+00       2489.95  0.000000E+00       2640.80       6723.92  0.000000E+00       11657.8       57.5218       9046.71 -1.000000E+10 -1.000000E+10 -1.000000E+10 -1.000000E+10 -1.000000E+10  0.000000E+00  0.000000E+00  0.000000E+00  0.000000E+00  0.000000E+00  0.000000E+00  0.000000E+00  0.000000E+00  0.000000E+00  0.000000E+00  0.000000E+00  0.000000E+00       Funtag_D
      71  4.403660E+05  4.002129E+05       40153.1       4.96459     0.0905976       1.31047      0.181912      0.228260    0.00992341    -0.0154132    0.00817576       1.01876       Opttag_D
      71       76.9446       1.00695       1.00000  0.000000E+00       1.00000  0.000000E+00      0.926321       72.7932       1.00000       33.5517  0.000000E+00       8.93779  0.000000E+00       8.93779  0.000000E+00  0.000000E+00      -17.8756       78.9652       3.74714        1       Scatag_D
      71       1877.54       5656.88  0.000000E+00  0.000000E+00       2489.69  0.000000E+00       2641.05       6728.83  0.000000E+00       11656.0       57.7574       9045.35 -1.000000E+10 -1.000000E+10 -1.000000E+10 -1.000000E+10 -1.000000E+10  0.000000E+00  0.000000E+00  0.000000E+00  0.000000E+00  0.000000E+00  0.000000E+00  0.000000E+00  0.000000E+00  0.000000E+00  0.000000E+00  0.000000E+00  0.000000E+00       Funtag_D
      72  4.403518E+05  4.001893E+05       40162.5       5.44889     0.0909053       1.30955      0.181899      0.228245    0.00998671    -0.0154055    0.00818290       1.01887       Opttag_D
      72       76.9471       1.00642       1.00000  0.000000E+00       1.00000  0.000000E+00      0.926304       72.7962       1.00000       33.5520  0.000000E+00       8.93800  0.000000E+00       8.93800  0.000000E+00  0.000000E+00      -17.8760       78.9652       3.74714        1       Scatag_D
      72       1880.82       5658.08  0.000000E+00  0.000000E+00       2489.33  0.000000E+00       2641.97       6736.61  0.000000E+00       11653.7       57.9462       9044.07 -1.000000E+10 -1.000000E+10 -1.000000E+10 -1.000000E+10 -1.000000E+10  0.000000E+00  0.000000E+00  0.000000E+00  0.000000E+00  0.000000E+00  0.000000E+00  0.000000E+00  0.000000E+00  0.000000E+00  0.000000E+00  0.000000E+00  0.000000E+00       Funtag_D
      73  4.403448E+05  4.001492E+05       40195.6       13.0388     0.0916871       1.30764      0.181878      0.228226     0.0100943    -0.0154381    0.00819408       1.01935       Opttag_D
      73       76.9534       1.00555       1.00000  0.000000E+00       1.00000  0.000000E+00      0.926265       72.8015       1.00000       33.5563  0.000000E+00       8.93796  0.000000E+00       8.93796  0.000000E+00  0.000000E+00      -17.8759       78.9652       3.74714        1       Scatag_D
      73       1885.96       5663.41  0.000000E+00  0.000000E+00       2488.77  0.000000E+00       2643.72       6762.02  0.000000E+00       11650.6       58.0083       9043.10 -1.000000E+10 -1.000000E+10 -1.000000E+10 -1.000000E+10 -1.000000E+10  0.000000E+00  0.000000E+00  0.000000E+00  0.000000E+00  0.000000E+00  0.000000E+00  0.000000E+00  0.000000E+00  0.000000E+00  0.000000E+00  0.000000E+00  0.000000E+00       Funtag_D
      74  4.403258E+05  4.001487E+05       40177.2       4.72459     0.0918128       1.30745      0.181876      0.228226     0.0100957    -0.0154642    0.00819023       1.01925       Opttag_D
      74       76.9544       1.00555       1.00000  0.000000E+00       1.00000  0.000000E+00      0.926258       72.8029       1.00000       33.5574  0.000000E+00       8.93784  0.000000E+00       8.93784  0.000000E+00  0.000000E+00      -17.8757       78.9652       3.74714        1       Scatag_D
      74       1884.19       5662.35  0.000000E+00  0.000000E+00       2488.73  0.000000E+00       2643.46       6748.21  0.000000E+00       11650.5       57.9067       9041.76 -1.000000E+10 -1.000000E+10 -1.000000E+10 -1.000000E+10 -1.000000E+10  0.000000E+00  0.000000E+00  0.000000E+00  0.000000E+00  0.000000E+00  0.000000E+00  0.000000E+00  0.000000E+00  0.000000E+00  0.000000E+00  0.000000E+00  0.000000E+00       Funtag_D
      75  4.403181E+05  4.001486E+05       40169.5       3.89838     0.0919867       1.30711      0.181876      0.228228     0.0100961    -0.0155044    0.00818545       1.01924       Opttag_D
      75       76.9557       1.00557       1.00000  0.000000E+00       1.00000  0.000000E+00      0.926249       72.8040       1.00000       33.5595  0.000000E+00       8.93759  0.000000E+00       8.93759  0.000000E+00  0.000000E+00      -17.8752       78.9652       3.74714        1       Scatag_D
      75       1881.99       5662.25  0.000000E+00  0.000000E+00       2488.70  0.000000E+00       2642.94       6745.56  0.000000E+00       11650.6       57.7923       9039.73 -1.000000E+10 -1.000000E+10 -1.000000E+10 -1.000000E+10 -1.000000E+10  0.000000E+00  0.000000E+00  0.000000E+00  0.000000E+00  0.000000E+00  0.000000E+00  0.000000E+00  0.000000E+00  0.000000E+00  0.000000E+00  0.000000E+00  0.000000E+00       Funtag_D

   MaxLik Scaling Cycle 0082

   Wilson plot

   Statistics in resolution bins after refinement cycle 0003
```


|  |  |
| --- | --- |
|  | - Rxpct factors plot- Rxpct factors table- <Log-Lik Gain>- Ln Sigma A- Correlation Coefficients |


```
   Iters     Tot_Funct    Xray_Funct    Geom_Funct          Grms     rmsD2init     maxD2init             R         Rfree           LLG       LLGfree     rms(bond)    rms(angle)
   Iters             K   B_IMPF_FRAG        K_MISS   B_TEMP_MISS   K_IMPF_MISS   B_IMPF_MISS        K_SOLV   B_TEMP_SOLV   K_IMPF_SOLV   B_IMPF_SOLV        B_TEMP     B_TEMP_11     B_TEMP_12     B_TEMP_22     B_TEMP_13     B_TEMP_23     B_TEMP_33      K_WILSON      B_WILSON      FGCYCLES
   Iters          BOND         ANGLE      IMPROPER       TORSION    SINTORSION      TRIGONAL        PLANAR  BCORRELATION       CONTACT     IDEALDIST        CHIRAL    SIMILARITY       OCCSSUM  UTILDISTANCE     UTILANGLE   UTILTORSION        RHOFIT        ADP-01        ADP-02        ADP-03        ADP-04        ADP-05        ADP-06        ADP-07        ADP-08        ADP-09        ADP-10      RHOFITPC            QM
      76  4.403091E+05  4.001435E+05       40165.6       4.44308     0.0923619       1.30596      0.181873      0.228230     0.0101097    -0.0155847    0.00818276       1.01930       Opttag_D
      76       76.9586       1.00549       1.00000  0.000000E+00       1.00000  0.000000E+00      0.926229       72.8059       1.00000       33.5640  0.000000E+00       8.93725  0.000000E+00       8.93725  0.000000E+00  0.000000E+00      -17.8745       78.9652       3.74714        1       Scatag_D
      76       1880.75       5662.88  0.000000E+00  0.000000E+00       2488.61  0.000000E+00       2642.39       6745.78  0.000000E+00       11650.3       57.7220       9037.22 -1.000000E+10 -1.000000E+10 -1.000000E+10 -1.000000E+10 -1.000000E+10  0.000000E+00  0.000000E+00  0.000000E+00  0.000000E+00  0.000000E+00  0.000000E+00  0.000000E+00  0.000000E+00  0.000000E+00  0.000000E+00  0.000000E+00  0.000000E+00       Funtag_D
   Geometry Restraint Deviations SCREEN Information

 
 No -bcorrel_zeroocchydrogens_factor flag found - keeping default: will set B for OCC=0 hydrogens to    1.200 times heavy-atom B


 TLS information. Total number of groups:     2


 Group   Natoms    FixRB   FixTLS   Group Tag    Group Spec
     1     2770      Y        Y     tls1    { A|* }
     2     2770      Y        Y     tls2    { B|* }
 
NOTE BUSTER_TLS_SET tls1 { A|* }
NOTE BUSTER_TLS_T   tls1     -1.00387E-01 -1.94646E-02 -1.04862E-01 -2.75119E-02  4.61084E-02  5.22733E-03
NOTE BUSTER_TLS_L   tls1      1.43977E+00  1.66678E+00  8.28191E-01  2.91853E-01 -4.93436E-01  3.60273E-01
NOTE BUSTER_TLS_S   tls1      2.45347E-01 -2.02592E-01  2.44710E-01 -1.91009E-01 -7.07893E-02 -2.32867E-01 -5.33582E-02 -7.54950E-02
NOTE BUSTER_TLS_O   tls1     -2.34016E+01 -8.37990E+01  1.17779E+01
NOTE BUSTER_TLS_FIX tls1 ALL
 
NOTE BUSTER_TLS_SET tls2 { B|* }
NOTE BUSTER_TLS_T   tls2     -1.34545E-01 -7.70270E-02  4.06613E-02  2.21513E-02  4.79797E-02 -1.95693E-02
NOTE BUSTER_TLS_L   tls2      1.12658E+00  1.16836E+00  9.36760E-01  5.85777E-01 -6.91255E-01 -5.92143E-01
NOTE BUSTER_TLS_S   tls2     -4.81861E-02  5.31306E-02 -8.29736E-02  4.54205E-02  2.18779E-01  5.84247E-02 -8.79871E-02  4.73445E-02
NOTE BUSTER_TLS_O   tls2      2.21707E+01 -7.20650E+01  3.37962E+00
NOTE BUSTER_TLS_FIX tls2 ALL

 For final position, Geometry function summary
     term                           number     weight       rms        GooF      function     fn/numb
     ================================================================================================
     BOND bond lengths (angs)       5655       2.000      0.0082       0.408      1880.75       0.333
     ANGL bond angles (degs)        7674       2.000        1.02       0.607      5662.88       0.738
     TORS split-harmonic tors       1270       0.000       18.10       1.139         0.00       0.000
     SINTOR sinusoidal tors         1270       2.000       18.10                  2488.61       1.960
     PLAN general plane (angs)       963       5.000      0.0148       0.741      2642.39       2.744
     BCORrelations (angs^2)         5655      10.000       0.968       0.345      6745.78       1.193
     CONT (bad contacts)               0       5.000                                 0.00
     IDEAL(ideal-dist contact)      5464       4.000                             11650.29
     CHIRAL (gelly semiharmon)       740       5.000                                57.72
     SIM similarity restraint for NCS and/or TARGET                               9037.22
     OCCSUM restraint on sum of occupanies                                           0.00       as none defined
     DISTAN utility restraints interatomic distance                                  0.00       as none defined
     UTILANGLE utility angle restraints                                              0.00       as none defined
     UTILTOR utility restraints on torsion angles                                    0.00       as none defined
     Peptide omega torsion angles                           2.99 degs - monitor only

 Weighted rms      0.0082         1.019  (temporary rms bond and angle for BUSTER to pick up!)
 Weighted intra-residual rms(bond) for StdProtein (excluding H)      0.0080
 Weighted intra-residual rms(bond) for Buffer     (excluding H)      0.0029
 Outputing geometry progress graph in plotmtv format to file:Geom_Res.mtv

 ############### Running final geometry/screen analysis (screen_final.txt)

 s/r gelly normal termination
```

Statistics and scale factors vs. cycle number
```

|  |  |  |
| --- | --- | --- |
|  | - Rxpct factors- <Log-Lik Gain>- GEOM Residuals- Ln Sigma_A_ | - Scale Factors- B Factors- Imperfection Factors |

```
 Writing F_calc(model+solvent) to complete.mtz

   Final Geometry Information


---


### 2. Final results

Statistics and scale factors vs. cycle number
```

|  |  |  |
| --- | --- | --- |
|  | - Rxpct factors- <Log-Lik Gain>- GEOM Residuals- Ln Sigma_A_ | - Scale Factors- B Factors- Imperfection Factors |

```
   Average Figure of Merit
   Structure Factor Amplitudes


 # normal termination

  Program stopped.
```

closesok message: time spent in tree = 982.00 (user) 34.05 (sys)

---

# Normal termination

```
 Resources usage summary:
     Time spent in client ...................   982.00 (user)       34.07 (system)
     Time spent in scripts (    27 calls) ...     1.67 (user)        0.12 (system)
     Time spent in server ...................     0.00 (user)        0.01 (system)

     Max resident memory used (kb) .......... 454048
     Number of page faults .................. 0 (major)    474784 (minor)
     Number of context switches ............. 6339 (niced)    4049 (other)
```
